# Persistent and reversible solid iodine electrodeposition in nanoporous carbons

**DOI:** 10.1038/s41467-020-18610-6

**Published:** 2020-09-24

**Authors:** Christian Prehal, Harald Fitzek, Gerald Kothleitner, Volker Presser, Bernhard Gollas, Stefan A. Freunberger, Qamar Abbas

**Affiliations:** 1 Institute for Chemistry and Technology of Materials, Graz University of Technology, Stremayrgasse 9, 8010 Graz, Austria; 2 Graz Centre for Electron Microscopy, Steyrergasse 17, 8010 Graz, Austria; 3 Institute of Electron Microscopy and Nanoanalysis, NAWI Graz, Graz University of Technology, Steyrergasse 17, 8010 Graz, Austria; 4 INM - Leibniz Institute for New Materials, Campus D2 2, 66123 Saarbrücken, Germany; 5 Department of Materials Science and Engineering, Saarland University, Campus D2 2, 66123 Saarbrücken, Germany; 6 IST Austria (Institute of Science and Technology Austria), Am Campus 1, 3400 Klosterneuburg, Austria; 7 Institute of Chemistry and Technical Electrochemistry, Poznan University of Technology, Berdychowo 4, 60-965 Poznan, Poland; 8 Present Address: Department of Information Technology and Electrical Engineering, ETH Zürich, Gloriastrasse 35, 8092 Zürich, Switzerland

**Keywords:** Batteries, Batteries, Supercapacitors, Batteries, Electrochemistry

## Abstract

Aqueous iodine based electrochemical energy storage is considered a potential candidate to improve sustainability and performance of current battery and supercapacitor technology. It harnesses the redox activity of iodide, iodine, and polyiodide species in the confined geometry of nanoporous carbon electrodes. However, current descriptions of the electrochemical reaction mechanism to interconvert these species are elusive. Here we show that electrochemical oxidation of iodide in nanoporous carbons forms persistent solid iodine deposits. Confinement slows down dissolution into triiodide and pentaiodide, responsible for otherwise significant self-discharge via shuttling. The main tools for these insights are in situ Raman spectroscopy and in situ small and wide-angle X-ray scattering (in situ SAXS/WAXS). In situ Raman confirms the reversible formation of triiodide and pentaiodide. In situ SAXS/WAXS indicates remarkable amounts of solid iodine deposited in the carbon nanopores. Combined with stochastic modeling, in situ SAXS allows quantifying the solid iodine volume fraction and visualizing the iodine structure on 3D lattice models at the sub-nanometer scale. Based on the derived mechanism, we demonstrate strategies for improved iodine pore filling capacity and prevention of self-discharge, applicable to hybrid supercapacitors and batteries.

## Abstract (web summary)

Iodide based energy storage is a potential candidate to improve performance of hybrid supercapacitors and batteries. Here, the authors revisit the previous understanding and show that electrochemical oxidation of iodide results in solid iodine deposits stabilized by the confinement of nanoporous carbons.

## Introduction

Reducing the ecological and economic footprint of electrochemical energy storage requires battery and storage concepts beyond standard intercalation-type Li-ion batteries. Current technologies suffer from the need of expensive, toxic, and flammable materials that are often obtained under harsh environmental and socioeconomic conditions^1^. Amongst the possible alternatives, iodine-based aqueous systems, such as iodide hybrid supercapacitors^2–5^, zinc iodine batteries^6–8^, or zinc iodide flow batteries^9,10^ are highly promising considering their performance, sustainability, and environmental aspects. However, to see their more widespread use in mobile or stationary applications, energy density, rate capability, and long-term stability need to become competitive with current Li-ion battery technology.

The critical process determining the performance of both aqueous^2,8,11^ and organic^12–15^ iodine-based energy storage is the reversible oxidation/reduction of iodide/iodine at 0.54 V vs. standard hydrogen electrode (SHE). At a planar platinum surface, triiodide (I_3_^−^) forms by electrochemically oxidizing iodide (I^−^) to iodine (I_2_), followed by comproportionation of I^−^ and I_2_^16,17^. In aqueous hybrid supercapacitors and iodine batteries, the reaction occurs in the nanopores of positively polarized carbon electrodes^2–4^. Confinement in such electrodes may change rates of individual reaction steps and hence the stability of I_2_ and I_3_^−^. While the polyiodides I_3_^−^ and I_5_^−^ are generally accepted to form during I^−^ oxidation, described mechanisms are ambiguous^2,3,7,8,18–20^. Works on carbon-iodine battery cathodes^15,21^ suggest that the physico-chemical mechanism during iodide/iodine oxidation/reduction is similar in both organic and aqueous electrolytes. The rather slow self-discharge of iodine-based electrochemical energy storage devices is currently attributed to immobile I_3_^−^ and I_5_^−^ confined in the narrow carbon pores of below 1 nm diameter^7,8,18^. However, given the known reaction mechanism on planar platinum electrodes^16,17^ and the use of battery electrodes with physically impregnated I_2_^6,7^, alternative electrodeposition of solid I_2_ in carbon nanopores ought to be considered.

Resolving the mechanism requires in situ techniques with chemical and structural sensitivity for all involved species in the nanoporous carbon. In situ Raman spectroscopy probes species in electrochemical cells^22–24^ and is sensitive to polyiodides (I_3_^−^, I_5_^−^)^25–28^. In situ small and wide-angle X-ray scattering (SAXS/WAXS) is sensitive to concentration changes and structural arrangements of molecules and ions in the nanoporous system^29–32^. Given the high scattering power of all iodine species (I^−^, I_3_^−^, I_5_^−^, I_2_), in situ SAXS/WAXS data should provide rich kinetic and structural information. However, the complexity induced by the multiphase character of these systems makes the SAXS data analysis highly challenging^29^.

Here, we show that I^−^ oxidation in microporous carbon (pore size <2 nm) produces solid I_2_, which can reach pore fillings of at least 30% and which in the iodide electrolyte partly dissolves into I_3_^−^ and I_5_^−^. The latter are responsible for self-discharge via shuttling. In situ Raman and ex situ ultraviolet–visible (UV–vis) spectroscopy data confirm I_2_, I_3_^−^, and I_5_^−^ during positive polarization and their reduction into I^−^ during negative polarization. In situ SAXS/WAXS data show that most of these species is confined to the carbon nanopores. Combined with stochastic modeling, in situ SAXS quantifies the amount of solid iodine deposit and visualizes its structural evolution in the pores. Based on the derived reaction mechanism, we show that high capacity with low self-discharge requires a small concentration of mobile polyiodides and a large fraction of immobile iodine deposits.

## Results

In situ Raman and SAXS/WAXS were done in two-electrode hybrid supercapacitor cells comprising activated carbon (AC) electrodes and aqueous 1 M NaI electrolyte. The iodide/iodine redox reaction takes place at the positive electrode, while the negative electrode stores charge exclusively via electrical double-layer capacitance. Faradaic capacity (current) at the positive electrode would be limited by the capacitance (current) of an equally sized negative electrode^33^. We increased the amount of iodine within the positive electrode by oversizing the negative AC electrode about 10-times in mass to exploit the Faradaic capacity and enhance the in situ Raman and SAXS/WAXS signals. The microporous AC has a mean pore size of 0.81 nm, a specific surface area of 1763 m^2^ g^−1^, and a specific pore volume of 0.84 cm^3^ g^−1^ (Supplementary Table 1 and Supplementary Fig. 1). The experimental set-ups and cell assemblies are discussed in the Methods and sketched in Supplementary Fig. 2.

### In situ Raman spectroscopy

**Fig. 1:**
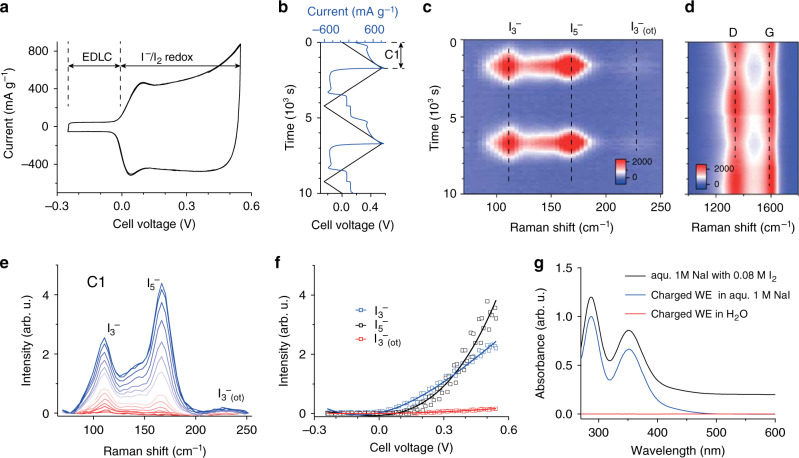
In situ Raman and ex situ UV–vis spectroscopy. **a** Current voltage curve of the asymmetric in situ Raman cell at a scan rate of 0.32 mV s^−1^. EDLC = region of electrical double-layer capacitance as the predominant charge-storage process. **b** Cell voltage and current versus time for two full cycles. **c**, **d** The corresponding Raman intensity as a function of time and Raman shift to track I_3_^−^ and I_5_^−^ bands (**c**), as well as the activated carbon (AC) G- and D-bands (**d**). **e** Raman spectra recorded during the first positive voltage sweep (C1). Colors change from red to blue with increased voltage. **f** I_3_^−^ and I_5_^−^ band intensities as a function of cell voltage. Solid lines represent third order polynomial fits to serve as a guide to the eye. **g** UV–vis absorption spectra of a 1 M NaI solution with 0.08 M I_2_ (black), 1 M NaI (blue), and deionized H_2_O where the washed, positively polarized (charged) carbon working electrode was soaked (red). Spectra are offset for clarity. More details and photographs of the measured solutions are given in Supplementary Fig. 5.

In situ Raman data were measured during cell voltage sweep between −0.25 V and +0.55 V at a scan rate of 0.32 mV s^−1^ (Fig. 1a). At negative voltages, the cell shows pure double-layer capacitance, with a current limited by the capacitance of the positive electrode. Upon positive polarization, I^−^ is oxidized at the positive electrode with a significantly enhanced current, which is limited by the capacitive current of the oversized negative electrode. The negative current of virtually the same magnitude during negative polarization above 0 V indicates a high coulombic efficiency of the reversible Faradaic iodide/iodine reaction.

The Raman intensity as a function of time for two voltage cycles shows fully reversible formation/disappearance of I_3_^−^ and I_5_^−^ at Raman shifts of 110 cm^−1^ and 165 cm^−1^, respectively (Fig. 1b, c)^26,27^. The band forming around 224 cm^−1^ is an overtone of the I_3_^−^ band^28^. At larger Raman shifts, changes in the carbon-related G- and D-band intensity (band width and position) can be observed (Fig. 1d, Supplementary Fig. 3). With increasing cell voltage, the widths of the G- and D-modes increase, while they are slightly red- and blue-shifted, respectively and the *I*_D_-to-*I*_G_ ratio is lowered. This can be explained by a superposition of charge transfer and an increase of defects^34–37^, which is qualitatively consistent with ex situ results reported earlier^18,38^.

As positive polarization proceeds, initially dominant I_3_^−^ band intensity gives way to the dominance of I_5_^−^ band intensity (Fig. 1e, f, curves from red to blue). Note that the I_3_^−^ and I_5_^−^ band intensities do not directly correlate with their concentration, since the scattering cross-sections of the polyiodides may vary significantly (resonant vs. non-resonant scattering)^39^. To quantify the Raman band intensity changes, we deconvolute the Raman spectra as shown in Supplementary Fig. 4^27^. The band intensities as a function of the cell voltage (Fig. 1f) show an increasing I_5_^−^ to I_3_^−^ band intensity ratio with increasing cell voltage. Hence, relative to I_3_^−^, I_5_^−^ forms at accelerated rates at higher cell voltages.

To check whether I_2_ had formed (next to I_3_^−^ and I_5_^−^), we soaked charged (positively polarized) and washed AC electrodes for several minutes in either pure water or 1 M NaI and recorded ex situ UV-vis spectra (Fig. 1g, details in Methods). While pure water would leave I_2_ confined to the AC nanopores, it should be dissolved in the 1 M NaI solution via ${\mathrm{I}}_2 + {\mathrm{I}}^ - \rightleftharpoons {\mathrm{I}}_3^ -$. Indeed, the 1 M NaI solution turned brownish, while pure water remained colorless (Supplementary Fig. 5). The UV–vis spectra in Fig. 1g confirm a large quantity of I_3_^−^ in the 1 M NaI solution and hence the presence of confined I_2_ in the charged positive AC electrode^40^. As an independent proof, we detected iodine after immersing the charged positive AC electrode for several minutes in ethanol, which directly dissolves I_2_ (Supplementary Fig. 5)^41^.

### In situ small and wide angle X-ray scattering

**Fig. 2:**
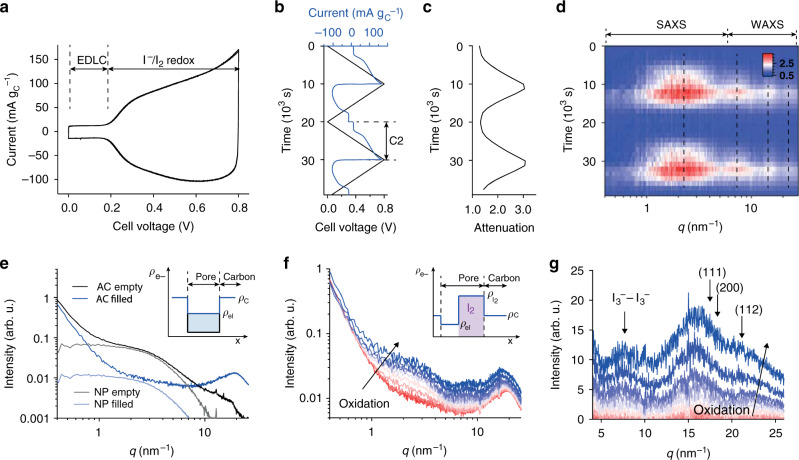
In situ small and wide-angle X-ray scattering. **a** Current voltage curve of the asymmetric in situ SAXS/WAXS cell at a scan rate of 0.08 mV s^−1^. **b** Current and cell voltage as a function of time for two full cycles. **c** The corresponding X-ray attenuation (negative logarithm of X-ray transmission^42^) versus time. **d** Relative scattering intensity change as a function scattering vector length *q* and time. **e** Ex situ SAXS/WAXS intensity versus scattering vector length *q* of the empty activated carbon electrode and the same electrode filled with electrolyte. Nanopore (NP) scattering intensities, obtained by subtracting the particle scattering intensity and the electrolyte structure factor (Supplementary Fig. 8), are given in gray and light blue. The inset schematically shows the electron (scattering length) density contrast between carbon skeleton and pore. **f** SAXS/WAXS intensities versus scattering vector length *q* during positive polarization. Color changes from red to blue with increasing cell voltage. The inset schematically shows electron (scattering length) density contrasts between carbon skeleton, electrolyte-filled pore and solid (crystalline) iodine. The scattering length densities of iodine, carbon and electrolyte are assumed with 3.49 × 10^11^ cm^−2^, 1.61 × 10^11^ cm^−2^, and 1.01 × 10^11^ cm^−2^, respectively. **g** The corresponding WAXS intensities during positive polarization, after subtracting the electrolyte structure factor contribution at 0 V cell voltage. I_2_ crystal diffraction peak positions are indicated, (111) at 25.1 nm^−1^, (200) at 29.0 nm^−1^, and (220) at 41.0 nm^−1^.

In situ Raman and ex situ UV–vis provide evidence for the formation of polyiodides (I_3_^−^, I_5_^−^) and I_2_ during I^−^ oxidation. However, these methods fail to quantify the absolute amount of the species, to locate them in the nanopores, and to clarify whether I_2_ is a solute or solid. In situ SAXS/WAXS can afford all this (Fig. 2).

As before, we use an asymmetric two-electrode cell with oversized negative electrode and ran cell voltage sweeps with the scan rate controlled by the total cell voltage. The cell exhibits pure double-layer capacitance limited by the capacitance of the positive AC electrode at cell voltages below +0.2 V (Fig. 2a). Above this voltage, I^−^ is oxidized at the positive AC electrode, leading to a significantly enhanced current (in line with electrochemical data of the in situ Raman measurements). The X-ray transmission of the electrolyte-filled positive AC electrode was simultaneously recorded by a photodiode placed behind the in situ cell, and quantifies the material flux in and out of the positive AC electrode (Fig. 2c). The significant increase of the X-ray attenuation (details in ref. ^42^) indicates the accumulation of large amounts of iodide, iodine, and polyiodides within the AC pores. The relative scattering intensity as a function of time follows the applied voltage and resulting current (Fig. 2b, d). During positive polarization, the scattering intensity in the SAXS regime at *q* < 5 nm^−1^ and in the WAXS regime has a clear maximum at the peak voltage.

Before analyzing the scattering intensity changes during positive cell polarization, we first must consider the data from the ex situ SAXS of the empty and electrolyte filled nanoporous carbon (Fig. 2e). The scattering intensity of the nanoporous carbon can be split into three additive terms: one accounting for spatial correlations on the molecular level, called structure factor, a second one accounting for the spatial correlations of the nanopores, which we refer to as nanopore scattering, and a third one accounting for the scattering contribution of the AC particles (with a size around 1 µm)^31,43,44^. Given the reciprocal relationship between the size of real-space objects and their appearance on the scattering curve, the structure factor contribution is most prominent at large scattering vectors *q* (large scattering angles). The nanopore scattering appears as a distinct intensity hump at intermediate scattering vectors *q*, with the shape and the position containing information about the pore morphology, size distribution, and mean pore size. The increased scattering intensity at *q* < 0.8 nm^−1^ is caused by the scattering of the AC particles. The nanopore scattering intensity depends on the squared electron density difference between pores and carbon skeleton; therefore, filling of the nanopores with electrolyte leads to a significant decrease of the scattering intensity (Fig. 2e, inset). At the same time, the electrolyte structure factor increases the scattering intensity in the WAXS regime. The distinct intensity peak around 20 nm^−1^ can be attributed to the first water structure factor peak (Supplementary Fig. 6).

Qualitatively, the large SAXS/WAXS intensity increase at low and large scattering vectors *q* (Fig. 2f, red to blue for increasing polarization) can be assigned to different contributions. The distinct intensity hump around 3 nm^−1^ has a shape comparable to the nanopore scattering contribution of the empty AC shown in Fig. 2e. This can only be explained by large amounts of a high-density compounds accumulating in its characteristic nanopores. The electron densities *ρ* of carbon skeleton and electrolyte-filled nanopores (insert in Fig. 2f) point at the formation of solid iodine since dissolved polyiodides alone could not account for the necessary electron density increase.

In the WAXS regime (*q* > 6 nm^−1^), distinct intensity peaks form around 7.5 nm^−1^, 16 nm^−1^, and 21.5 nm^−1^ (Fig. 2g). To better visualize the peak formation, we subtracted the structure factor contribution of the 1 M NaI electrolyte (that is, the WAXS intensity at 0 V cell voltage). We attribute the peak around 7.5 nm^−1^ to I_3_^−^ – I_3_^−^ correlations of a highly concentrated triiodide solution in the cavities formed by nanoporous carbon and solid I_2_, justified by the mean distance between I_3_^−^ species being in the order of 2π/7.5 nm^−1^ = 0.84 nm. The peaks at 16 nm^−1^ and 21 nm^−1^ do not exactly match diffraction peaks of crystalline I_2_, indicating that solid I_2_ forms in a crystal structure slightly different from bulk I_2_, partially in an amorphous state and/or the confined nanocrystals are strongly distorted. The diffraction peak widths (FWHM), as obtained by Gaussian peak fitting (Supplementary Fig. 7), are located in the region of 10.5°–19° 2θ. According to Scherrer’s equation, this translates into a crystallite size between 0.55 – 0.9 nm, which fits well to the mean carbon pore size of 0.81 nm and the intensity changes found in SAXS.

Qualitatively, the in situ SAXS/WAXS and Raman data analysis provide correlating complementary information. Based on these data, we see that during oxidation, solid iodine particles are deposited in the carbon nanopores. At the same time, significant amounts of I_3_^−^ and I_5_^−^ are generated. The WAXS correlation peak at 7.5 nm^−1^ suggests a highly concentrated solution of polyiodides that is, to a large extent, confined to the nanopores. Solid iodine domains are likely to be present in crystalline form, since diffraction peak widths point at crystallite sizes up to 0.9 nm, in accord with the mean carbon pore size of 0.81 nm.

### Quantification of iodine phase evolution via stochastic modeling

To quantify the amount of the formed I_2_ in the nanopores, we first separate the electrolyte/carbon structure factor (high *q*) and the particle scattering (low *q*) from the pure nanopore scattering (intermediate *q*). The separation procedure is described in the Methods and Supplementary Fig. 8. In a second step, we use the concept of plurigaussian random fields to fit the reduced experimental in situ SAXS curves and generate 3D lattice models of the carbon nanopore structure filled with the solid iodine phase. This involves modeling the empty carbon nanopore structure using a Gaussian random field (GRF) *Y*(**x**) and the solid I_2_ via a second Gaussian random field *Z*(**x**). After the carbon nanopore structure has been obtained from a fit to the SAXS intensity of the empty carbon electrode (Supplementary Fig. 9 and ref. ^45^), the in situ SAXS data are fitted using the I_2_ pore occupancy and two structural parameters of the I_2_ phase as fit parameters. These are the correlation parameter *l*_*Z*_ of the GRF *Z*(**x**) and the parameter *δ* accounting for carbon-iodine correlations (Supplementary Fig. 10). A detailed description of the plurigaussian random field modeling and fitting is given in the Methods, limitations and sources of error in Supplementary Note 1.

**Fig. 3:**
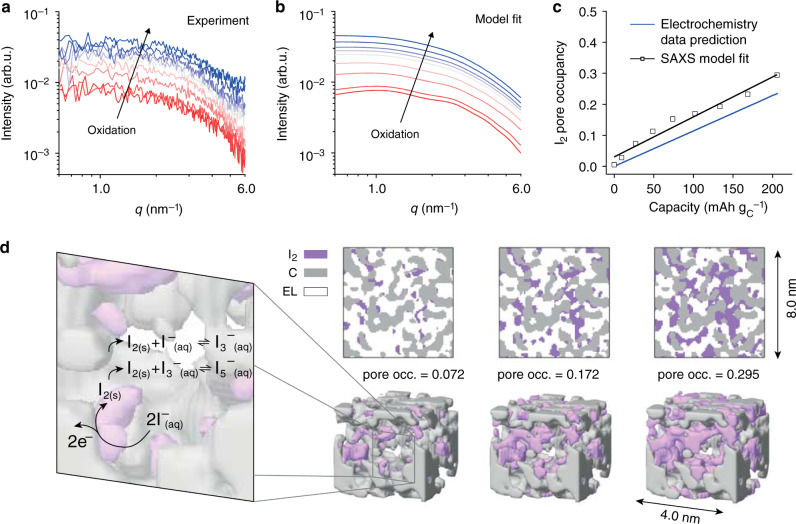
Reaction mechanism of I_2_ formation and quantification of phase evolution via stochastic modeling. **a** Reduced experimental in situ SAXS data during positive cell polarization (after subtraction of particle and electrolyte structure factor scattering contributions, see Supplementary Fig. 8). **b** Best-fit modeled small-angle scattering intensities versus scattering vector length *q* for fractions for an increasing fraction of iodine phase (from red to blue curves), using the concept of plurigaussian random fields (see Methods). **c** Iodine pore occupancy vs. specific capacity as obtained from the in situ SAXS data fits (black, solid line represents linear fit) and estimated from specific capacity and known electrode porosity. **d** 3D lattice models generated by plurigaussian random fields for increasing iodine pore occupancies (pore occ.). The cross-sections (top) and 3D cut-outs correspond to the modeled in situ SAXS data shown in **b**. The reaction mechanism derived from in situ Raman and in situ SAXS/WAXS data is shown schematically in the detail on the left.

The modeled in situ SAXS intensities fit well to the reduced experimental in situ SAXS intensities measured during positive polarization (Fig. 3a, b, I^−^ oxidation proceeds from red to blue). The increasing scattering intensity is caused by the increasing amount of solid iodine as visualized on cross-sections and 3D cut-outs in Fig. 3d (see Supplementary Video of the 3D model at maximum pore filling). The overall shape of the scattering curve remains similar to the shape of the empty nanopore structure (Fig. 2e and bottom red curve in Fig. 3a at the beginning of I^−^ oxidation). This finding is attributed to the similar characteristic feature size of the solid iodine structure and the empty carbon nanopores on the one hand (compare fit parameters *l*_*Z*_ and *l*_*Y*_, Supplementary Fig. 11), and the correlation between the iodine and carbon structure on the other hand (fit parameter *δ*). Given the size of the confining carbon pore structure and the crystallite sizes obtained from the Scherrer analysis (Supplementary Fig. 7), the iodine particles should not be much larger than the mean pore size of 0.81 nm. The I_2_ pore occupancy reaches a maximum of 30% at the maximum positive cell voltage of 0.8 V (Fig. 3c). This I_2_ pore occupancy fits well with the value estimated from Faradaic capacity, assuming that all capacity forms I_2_ (blue line; in reality, a certain fraction also reacts to polyiodides). The SAXS integrated intensity analysis in Supplementary Note 2 provides further (model-free) evidence that the I_2_ pore occupancies shown in Fig. 3c lie in the right range. Given the poor electronic conductivity of I_2_, the found high degrees of pore filling are remarkable. We conclude that solid I_2_ rather than dissolved polyiodides represent the primary, capacity-relevant fraction of oxidized species. While in situ Raman data confirms the existence of significant amounts of I_3_^−^ and I_5_^−^, relative to solid I_2_, their absolute amount is small at least up to charging capacities of 200 mAh g_C_^−1^.

The quantification via plurigaussian random fields, presented here, confirms the qualitative data interpretation from above. Contrary to the current state-of-the-art, oxidation of iodide in nanoporous carbon electrodes results in solid iodine nanoparticles, as well as dissolved I_3_^−^ and I_5_^−^ polyiodides. The physico-chemical mechanism to stabilize solid I_2_ is further discussed in Supplementary Note 3. A hysteresis in the iodine formation/dissolution as a function of the WE potential during charge/discharge suggests that solid I_2_ deposited in the porous carbon requires a significant overpotential to be dissolved (Supplementary Note 3). This is in line with the stabilization of solid I_2_ by severe carbon–iodine interactions in the nanoporous confinement.

Processes in the positive electrode of a hybrid NaI supercapacitor are comparable to those in common conversion-type battery electrodes, such as Li-S or Li-O_2_ battery cathodes, where solid Li_2_S or Li_2_O_2_ precipitate from solution species^46,47^. During charging and discharging, the solid active material is deposited within the nanopore network of a carbon cathode. The pore occupancy of active material hence determines achievable capacities and reversibility.

### The iodide formation reaction mechanism

The in situ and ex situ data allow deriving a detailed reaction mechanism of the (poly)iodide/iodine redox chemistry in nanopore confinement (Fig. 3d). Specifically, (i) in situ Raman, ex situ UV-vis and in situ SAXS (Figs. 1–3) confirm the formation of I_2_, I_3_^−^ and I_5_^−^ during I^−^ oxidation. (ii), I_5_^−^ generation is delayed with respect to I_3_^−^ upon oxidation process as seen in their Raman intensities (Fig. 1f–g). This indicates that larger amounts of generated I_3_^−^ and I_2_ accelerate I_5_^−^ formation. (iii), In situ SAXS/WAXS data confirm the formation of solid I_2_ nanoparticles or clusters in the nanopores.

At first, I^−^ is oxidized to I_2_ at the carbon-electrolyte interface (Eq. (1)). This reaction leads to solid I_2_ nanoparticles with a size limited by the electron tunneling/conduction thickness of the insulating I_2_ and the confining carbon cavities. Concurrently, I_2_ comproportionates to some extent with I^−^ to I_3_^−^ (Eq. (2)), with the amount of the latter growing with the amount of I_2_ (steadily increasing amounts of I_3_^−^ and I_2_, Figs. 1 and 3). As I_3_^−^ and I_2_ amounts grow in the nanopores, the I_5_^−^ formation via the comproportionation (Eq. (3)) accelerates.

1 $$2 {\mathrm{I}}^{-} _{\mathrm{(aq)}} \rightarrow {\mathrm{I}}_{2({\mathrm{s}})} + 2{\mathrm{e}}^{-}$$

2 $${\mathrm{I}}_{2({\mathrm{s}})} + {\mathrm{I}}_{({\mathrm{aq}})}^ - \, \rightleftharpoons \, {\mathrm{I}}_{3({\mathrm{aq}})}^ -$$

3 $${\mathrm{I}}_{2({\mathrm{s}})} + {\mathrm{I}}_{3({\mathrm{aq}})}^ - \, \rightleftharpoons \, {\mathrm{I}}_{5({\mathrm{aq}})}^ -$$

The direct electrochemical generation of I_3_^−^ via $3\,{\mathrm{I}}^ - \to {\mathrm{I}}_3^ - + 2\,{\mathrm{e}}^ -$ can be excluded since the further precipitation of I_2_ via Eq. (2) would require to first reach an I_3_^−^ concentration which would drive disproportionation; hence, I_3_^−^ would level off while I_2_ grows. Instead, in situ Raman and SAXS show steadily and concurrently evolving amounts of I_3_^−^ and I_2_. Based on the equilibrium constant of Eq. (2), the concentration of I_3_^−^ is orders of magnitudes higher than the I_2_ concentration at all times^40,41,48^. The generation of large amounts of I_2_ via precipitation (Eq. (2)) would require extremely high I_3_^−^ concentrations, which appears unlikely.

### Low self-discharge, high capacity density iodine energy storage

The detected amount of solid I_2_ produced in the carbon nanopores during voltage cycling is quite remarkable, with a specific capacity reaching 200 mAh g_C_^−1^. To gauge the maximum amount of iodine possible to be electrodeposited in the positive AC electrode, neither the negative AC electrode capacitance nor total I^−^ in the system must limit iodine electrodeposition. This is realized with a large, about 100 times oversized AC counter electrode and a large electrolyte volume (details in Methods, cyclic voltammetry in Supplementary Fig. 12).

**Fig. 4:**
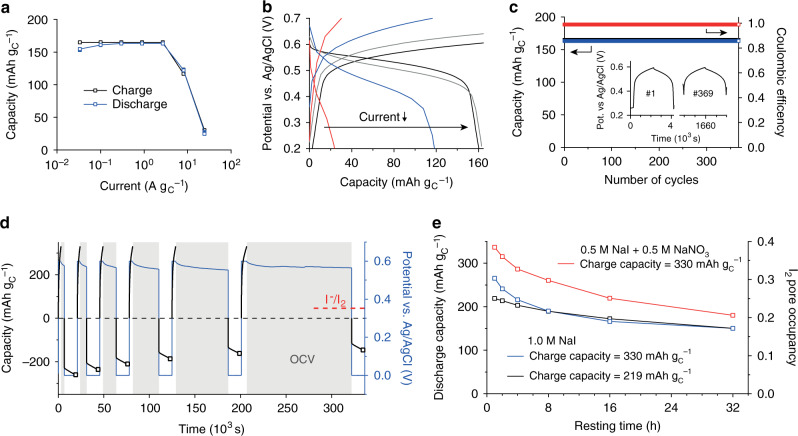
Low self-discharge, high capacity density iodine energy storage. **a** Specific capacity versus specific current for galvanostatic charge/discharge with capacity limitation of 160 mAh g_C_^−1^. **b** WE potential as a function of capacity for the four highest currents with 24.3 A g_C_^−1^ (red), 8.1 A g_C_^−1^ (blue), 2.7 A g_C_^−1^ (gray), 0.9 A g_C_^−1^ (black) of the same galvanostatic charge/discharge measurements. **c** Charge/discharge capacity and coulombic efficiency versus number of cycles at a rate of 0.3 A g_C_^−1^. The inset shows the WE potential as a function time for 2 cycles. **d** Potentiostatic charge / discharge (+0.6/0 V vs. Ag/AgCl) measurements with increasing resting times at open-circuit voltage (OCV) between the charging and discharging steps. Potentiostatic charge was limited to 330 mAh g_C_^−1^, potentiostatic discharge to 4 h within which the current always dropped to negligible values. The protocol is exemplified for the 1 M NaI electrolyte and a charging capacity of 330 mAh g_C_^−1^. **e** Discharge capacities vs. resting times for the protocol shown in **a** using the 1 M NaI electrolyte and two different charging capacities (219 mAh g_C_^−1^ and 330  mAh g_C_^−1^) and the 0.5 M NaI + 0.5 M NaNO_3_ electrolyte with a charging capacity of 330 mAh g_C_^−1^. The right axis shows the iodine pore occupancy, estimated from the discharge capacities and the known electrode porosity values.

The system shows an excellent rate performance as shown by galvanostatic charge/discharge measurements with a set capacity limit of 160 mAh g_C_^−1^ (Fig. 4a, b). Note that the capacity limit rather than the potential limit needs to be set for this system, since polyiodide shuttling between WE and CE at high capacities hinders a rise in the WE potential when all pores are occupied by I_2_. The cycle life is very high and the Coulombic efficiency is constantly close to 100% (Fig. 4c). There are no signs for performance degradation after cycling several hundred times. Both rate capability and cycle life are comparable to hybrid supercapacitors even though the stored energy is much higher.

To check for the maximum capacity that can be practically used in the positive AC electrode, we oxidized I^−^ at a constant potential of +0.6 V vs Ag/AgCl at the positive working electrode up to a certain charging capacity. After leaving the cell at open-circuit for different resting times (1–32 h), the remaining I_2_ was measured by a potentiostatic discharge to 0 V for 4 h (Fig. 4d).

The self-discharge increases as the charging capacity of the positive AC electrode is increased (Fig. 4e and Supplementary Fig. 13). Independent of the positive electrode loading, already after a few hours resting time, the discharge capacities approached a similar level. This is also shown by charging to three different capacities (219 mAh g_C_^−1^, 330 mAh g_C_^−1^, and 441 mAh g_C_^−1^) and waiting for 4 h at OCV (Supplementary Fig. 13). After 32 h of resting, the discharge capacities reached a value of 150 mAh g_C_^−1^. Assuming that most capacity is stored in the form of solid I_2_ and by considering the known electrode porosities (Supplementary Table 1) this corresponds to an I_2_ pore occupancy of 0.17.

The proposed reaction mechanism explains the increasing self-discharge with increasing amounts of iodine loading/charging capacities. The more iodine is deposited, the more mobile I_3_^−^ and I_5_^−^ are formed chemically. Owing to their negative charge, a certain fraction of polyiodides will remain adsorbed in the nanopores of the positively polarized WE. The more polyiodides are generated, the higher is also their fraction that diffuses to the CE, where they are reduced to I^−^ to promote self-discharge. Hence, future strategies to increase the iodine pore occupancy and reduce self-discharge should focus on reducing the concentration of shuttling polyiodides. Based on the reaction mechanism in Fig. 3d (Eqs. (2) and (3)), this involves reducing the I^−^ concentration by, for example, using additional, electrochemically inert anions like NO_3_^−^, which allow precipitating a larger fraction of the I^−^ without anion starvation. Using aqueous 0.5 M NaI and 0.5 M NaNO_3_ as electrolyte (red curve, Fig. 4e), I_2_ pore filling and discharge capacities improve significantly. This not only demonstrates a practical way towards improved energy densities of carbon-iodine electrodes used in hybrid supercapacitors or batteries, but also independently confirms the reaction mechanism, derived from in situ Raman and SAXS/WAXS.

## Discussion

In situ Raman and in situ SAXS/WAXS have shown that oxidation of I^−^ in the confinement of carbon nanopores generates not only I_3_^−^ and I_5_^−^ polyiodides, but to a large extend solid iodine nanoparticles. Induced by the confinement of the carbon nanopores, solid I_2_ dissolves only slowly during self-discharge. The derived reaction mechanism explains all recorded in situ spectroscopic, in situ scattering, and electrochemical data and suggests pathways to increase capacity densities and further reduce self-discharge. Electrochemical measurements evidence remarkably high capacities. In terms of specific and volumetric capacity solid I_2_ (211 mAh g^−1^, 1040 mAh cm^−3^) surpasses todays benchmark Li-ion cathode materials, such as LiFePO_4_ (170 mAh g^−1^, 596 mAh cm^−3^). To make capacity values comparable to intercalation-type cathode materials the total capacity of the iodine system needs to be normalized by the mass or volume of carbon, micropore electrolyte and deposited I_2_. An I_2_ pore occupancy of 40% in our carbon with 60% porosity would result in practical capacities of 108.5 mAh g^−1^ and 250 mAh cm^−3^. We show how to achieve such high packing densities at rates significantly exceeding those of Li-ion cathode materials. The absence of slow intercalation processes enables rates comparable to hybrid supercapacitors. An effective strategy to improve the iodine pore filling capacities lies in reducing the concentration of iodide in the carbon nanopores in the charged state using an inert supporting electrolyte. Further performance increase relies on systematic parameter studies varying the nanopore structure of the used carbons, the electrolyte volume, salt concentration, and the amount of supporting electrolyte.

Understanding that I^−^ oxidation in carbon nanopores electrodeposits solid iodine calls for a paradigm shift in two aspects. First, increasing the capacity of such systems relies on strategies to increase the pore filling with solid iodine. Second, avoiding self-discharge means reducing the concentration of mobile polyiodide species generated by comproportionation. Until now, confined immobile polyiodides were thought to be responsible for the excellent self-discharge properties of iodine-based energy storage.

Considering the shown strategies to improve capacity and self-discharge, the similarities with other conversion-type batteries, such as Lithium-sulfur, are remarkable. Carbon electrodes with a high degree of iodine pore filling are thus alternative, sustainable, and environmentally friendly conversion-type battery electrodes that may be used in combination with a supercapacitor counter-electrode (hybrid supercapacitor) or a battery anode material (e.g., zinc-iodine battery).

From a broader perspective, the work shows the necessity of applying advanced in situ techniques for detailed insights into electrochemical mechanisms and to derive practical conclusions for real devices. We show that properties and function of complex emerging systems for electrochemical energy storage are equally controlled by chemistry (in situ spectroscopy) and the nanoscale structure (in situ scattering). In combination with stochastic modeling, in situ SAXS/WAXS allowed for quantifying the iodine pore filling and visualizing the (sub-)nm iodine phase evolution during electrochemical cycling. Hence, in situ SAXS proves as a powerful experimental method to study the phase evolution of active materials in nanoporous carbons and beyond.

## Methods

### Materials

Free-standing carbon electrodes were prepared by mixing 90 mass% of the microporous KOH-activated carbon (MSP-20, Kansai Coke and Chemicals, Supplementary Fig. 1, Supplementary Table 1) with 5 mass% carbon black (C65 from Imerys), and 5 mass% of polytetrafluoroethylene (60% dispersion in water from 3 M Chemicals) in ethanol. After stirring at +70 °C, the obtained dough was pressed and rolled on a glass plate into a thin sheet. Disc electrodes were punched from the sheet and dried at 120 °C, resulting in a final thickness of about 150 μm. Sodium iodide (NaI, 99.5%) was purchased from Alfa-Aesar and dried at 110 °C overnight before preparing the aqueous 1.0 M NaI electrolyte with de-ionized water. The pH of the electrolyte was 6.5 and the conductivity 82 mS cm^−1^.

### Raman spectroscopy

In situ Raman spectroscopy was carried out using a LabRAM HR 800 spectrometer combined with an Olympus BX41 microscope. The spectra were measured from the top, with the focus plane underneath the standard microscope glass slip (covering the cell assembly) and the electrolyte film on top of the carbon electrode. A schematic drawing of the two-electrode electrochemical in situ cell ECC-Opto-Gas from EL-CELL (Hamburg, Germany) is given in Supplementary Fig. 2. As an objective lens a 40× Olympus LUCPlanFL N (NA = 0.6; corrected for the cover thickness) was used. To avoid sample damage, the laser with 532 nm wavelength worked at reduced power (0.5 mW). With the given grating/slit setup (300 mm^−1^; 200 µm) the spectral (pixel) resolution is about 3.6 cm^−1^. The acquisition time per spectrum was 30 s (4-fold accumulations) for a total measurement time and time resolution of 120 s/spectrum. In addition, the DuoScan System was applied to continuously scan the laser spot over a 20 × 20 µm area. With regard to the spectral analysis the band deconvolution of the I_3_^−^/I_5_^−^ bands (Supplementary Fig. 3) was done using three Lorentz peaks (initial position 110 cm^−1^, 143 cm^−1^, and 224 cm^−1^) and one Gaussian peak (initial position 165 cm^−1^), this routine is based on the band assignments from ref. ^27^. The deconvolution of the G- and D-band was done according to the method introduced in ref. ^34^.

### UV–vis spectroscopy

Positive carbon electrodes (mass = 3.0 mg) were charged in a two-electrode cell with oversized counter-electrode (mass = 34.0 mg) by voltage sweep (2.0 mV s^−1^) to a cell voltage of 1.0 V. After charging, the positive electrodes were taken out, washed for 1 min with deionized water and placed for 3 h in three different solutions: (i) deionized water, (ii) aqueous 1 M NaI and (iii) ethanol. UV-Vis absorption spectra of the solutions were recorded on a Cary 50 spectrophotometer (Varian).

### Small and wide-angle X-ray scattering

In situ SAXS/WAXS measurements were carried out with a custom-built two-electrode electrochemical in situ cell. The cell consisted of the positive AC electrode, a glass fiber separator (Whatman), a negative AC electrode and platinum foil current collector (CC). The assembly was sealed in a stainless-steel housing with adhesive film (Tixo) X-ray windows. To enhance the recorded signal by increasing the amount of oxidized I^–^ the negative electrode was oversized 8-times (mass = 32 mg, thickness = 600 µm, diameter = 12 mm) compared to the positive electrode (mass = 4 mg, thickness = 200 µm, diameter = 8 mm). Oversizing the negative electrode requires increasing its thickness rather than increasing the diameter to avoid detrimental, large ion diffusion pathways. To investigate only processes in the positive electrode, a hole with 2 mm diameter was cut into negative electrode, separator and CC.

SAXS/WAXS data were recorded on an in-house SAXS facility using Cu-Kα radiation and a 2D areal detector (Dectris Eiger R 1 M) with a nominal sample-to-detector distance of 100 mm. The primary 2D scattering data were azimuthally averaged and normalized by transmission values and recording time. Further, intensity data were multiplied by polarization $2/\left( {1 + (\cos 2\theta )^2} \right)$ and absorption $\left[ {1 - \tau ^{\frac{1}{{\cos 2\theta }} - 1}} \right]{\mathrm{/}}\left[ {{\mathrm{ln}}\tau - \frac{1}{{\cos 2\theta }}{\mathrm{ln}}\tau } \right]$ correction factors to adequately normalize scattering intensities at large scattering angles 2*θ*^49^. The background scattering intensity was recorded after the in situ SAXS measurements and removing the WE; and subtracted from in situ SAXS/WAXS data after primary data treatment as described above.

To separate the nanopore scattering contribution from the electrolyte structure factor and the low-*q* particle scattering, we chose a pragmatic approach: we first determined the electrolyte structure factor using a modified Porod fit of the form $I_{{\mathrm{porod}}} = P{\mathrm{/}}q^4 + A \cdot SF(q)$ at 6.5 nm^−1^ < *q* < 9 nm^−1^, where *SF*(*q*) is the electrolyte structure factor, *A* a multiplicative constant, and *P* the Porod constant. We assume that *SF*(*q*) corresponds approximately to the measured scattering intensity of the bulk electrolyte (as shown in Supplementary Fig. 8) and remains constant during cycling the cell. The parameters *A* and *P* were determined by the fit to the electrolyte filled AC electrode at zero cell voltage. After subtracting the electrolyte structure factor contribution $A \cdot SF(q)$, the particle scattering contribution was obtained from a power law fit of the form ${\mathrm{I}}_{{\mathrm{particle}}} = B{\mathrm{/}}q^{3.73} + C$ at 0.4 nm^−1^ < *q* < 1 nm^−1^, with *B* and *C* being the unknown fit parameters. The exponent of 3.73 was determined from a power law fit to the empty (bare) AC electrode. While $A \cdot SF(q)$ is assumed to be constant, the power law fit at low *q* with changing parameters *B* and *C* was carried out for all in situ SAXS intensities during cell polarization. The obtained particle scattering *B*/*q*^3.73^ was then subtracted from the experimental SAXS intensities.

The in situ SAXS data fits apply the concept of plurigaussian random fields^45^, as explained below. To ensure proportionality between experimental and modeled SAXS intensities we multiplied the modeled SAXS intensities by a factor *K*, given in Eq. (4). The constant *K* was determined once, by dividing the integrated intensity $\tilde I = {\int}_0^\infty {q^2} I\left( q \right){d}q$ of the experimental nanopore scattering intensity (with no iodine present) by the integrated intensity of the corresponding modeled SAXS intensity^45,50^. We assume the carbon nanopore structure and the corresponding GRF *Y*(**x**) to be constant during the in situ SAXS experiment. The GRF parameters of *Y*(**x**) are taken from a fit to the experimental ex situ SAXS intensity of the empty nanoporous carbon electrode (Supplementary Fig. 9 and Supplementary Table 2). The nanopore scattering intensity of the bare AC electrode was obtained by subtracting a constant background (determined via a standard Porod fit in the *q*-range 7 nm^−1^ < *q* < 9 nm^−1^) and the particle scattering contribution (determined by a power-law fit $I_{{\mathrm{power}}} = D{\mathrm{/}}q^\eta + E,$ for *q* < 1 nm^−1^) from the SAXS intensity of the empty AC electrode (solid black line). Since the nanopore scattering intensity is fitted with a simple two-phase model (see below), density fluctuations within the carbon matrix^51^ are neglected. We assumed a porosity of $1 - \phi _C = 60\%$ as obtained from gas sorption measurements (Supplementary Table 1), set the upper threshold value to ∞, and used *l*_*Y*_ and *d*_*Y*_ as fit parameters.

The in situ SAXS intensities (Fig. 3a) were fitted to three fit parameters: the I_2_ pore occupancy *ϕ*_*A*_, the correlation parameter *l*_*Z*_ of the iodine phase GRF (*Z*(**x**)), and the parameter *δ*, accounting for *Z*(**x**) (iodine) and *Y*(**x**) (carbon) correlations. The I_2_ pore occupancy values were constrained to values smaller than 0.35, estimated from electrochemical capacity values (Fig. 3c). The value *d*_*Z*_ was kept constant at a value of 30 nm (*d*_*Z*_ ≈ ∞). The scattering length densities of iodine, carbon and electrolyte were assumed with 3.49 × 10^11^ cm^−2^, 1.61 × 10^11^ cm^−2^, and 1.01 × 10^11^ cm^−2^, respectively. This corresponds to a mass density of 1.9 g∙cm^−3^ for the carbon skeleton, following previous works^43^, and 4.94 g cm^−3^ for iodine, corresponding to the iodine crystal density. The scattering length density of the electrolyte corresponds to a concentration of 1 M NaI in the aqueous solvent. To fit the in situ SAXS intensities, we minimized the sum of the squared residuum of all experimental SAXS intensities during a positive potential sweep. In other words, the parameters *l*_*Z*_ and *δ* were held constant while the iodine pore occupancy was used as fit parameter for each SAXS curve at its specific state of charge. The procedure was repeated for several values of *l*_*Z*_ and *δ* (Supplementary Fig. 11) and the parameter set with the minimum sum of squared residuum values taken as the solution.

### Plurigaussian random fields

We model the in situ SAXS data using the concept of plurigaussian random fields^45^. This allows retrieving 3D real space models of the solid iodine phase evolution within the carbon nanopore structure and quantifying the degree of iodine pore filling (Fig. 3). A detailed description of how plurigausian random fields are applied to in situ SAXS data of three-phase systems is given by Gommes et al.^45^.

The SAXS intensity *I*(*q*) as a function of the scattering vector length *q* is the Fourier transform of the electron density correlation function *C*(*r*)

4 $${{I}}\left( q \right) = K\int_0^\infty {C\left( r \right)} \frac{{\sin \left( {qr} \right)}}{{qr}}4\pi r^2dr,$$

with *K* being a constant that depends on the sample volume and instrumental parameters, such as detector efficiency. *C*(*r*) for a three-phase system consisting of phases A, B, C can be written as

5 $$C\left( r \right) = 	\, \left( {\rho _{\mathrm{A}} - \rho _{\mathrm{B}}} \right)\left( {\rho _{\mathrm{A}} - \rho _{\mathrm{C}}} \right)\left[ {P_{{\mathrm{AA}}}\left( r \right) - \phi _{\mathrm{A}}^2} \right]\\ 	+ \, \left( {\rho _{\mathrm{B}} - \rho _{\mathrm{A}}} \right)\left( {\rho _{\mathrm{B}} - \rho _{\mathrm{C}}} \right)\left[ {P_{{\mathrm{BB}}}\left( r \right) - \phi _{\mathrm{B}}^2} \right]\\ 	+ \, \left( {\rho _{\mathrm{C}} - \rho _{\mathrm{A}}} \right)\left( {\rho _{\mathrm{C}} - \rho _{\mathrm{B}}} \right)\left[ {P_{{\mathrm{CC}}}\left( r \right) - \phi _{\mathrm{C}}^2} \right].$$

Here, *ρ*_i_ is the electron density, *ϕ*_i_ the volume fraction and *P*_ii_(*r*) the two-point correlation function of phase i.

Before modeling the iodine phase evolution, we generate a 3D model of the empty nanoporous carbon structure using a fit to the ex situ SAXS curve of the empty carbon electrode and the concept of clipped Gaussian random fields. Detailed descriptions of the procedure are given in refs. ^31,43,50,52^. In brief, a Gaussian random field *Y*(***x***) is the sum of cosine waves with wave vectors distributed according to their power spectral density *f*_*Y*_(*k*) and phase factors *φ*_i_ randomly distributed between 0 and 2*π*^45,50,53,54^.

6 $$Y\left( {\mathbf{x}} \right) = \sqrt {\frac{2}{N}\mathop {\sum}\limits_{i = 1}^N {{\mathrm{cos}}} } ({\mathbf{k}}_{\mathbf{i}} \cdot {\mathbf{x}} - \varphi _{\mathrm{i}})$$

A suitable analytic two-point correlation function of the GRF is ref. ^31^

7 $$g_Y\left( r \right) = \frac{1}{{{\mathrm{cosh}}(r{\mathrm{/}}l_Y)}} \frac{{{\mathrm{sin}}(2\pi r{\mathrm{/}}d_Y)}}{{(2\pi r{\mathrm{/}}d_Y)}},$$

where *l*_*Y*_ is a correlation parameter related to the mean size of the pores and *d*_*Y*_ a parameter accounting for ordering effects in-between pores. Equation S4 translates into the following analytic expression for the power spectral density:

8 $$f_Y\left( k \right) = \frac{k}{\pi }l_Yd_Y\frac{{{\mathrm{sinh}}\left( {\pi kl_Y{\mathrm{/}}2} \right) {\mathrm{sinh}}(\pi ^2l_Y{\mathrm{/}}d_Y)}}{{\cosh \left( {\pi kl_Y} \right) + {\mathrm{cosh}}(2\pi ^2l_Y{\mathrm{/}}d_Y)}}.$$

To generate a two-phase porous structure from the GRF, we define threshold values *α* and *β* for the Gaussian distributed *Y*(***x***) values. All spatial coordinates ***x*** with *α* < *Y*(***x***) ≤ ∞ are assigned to the pore space; all other coordinates to the carbon skeleton. The two threshold values are related to the carbon volume fraction *ϕ*_C_ via:

9 $$\phi _{\mathrm{C}} = \frac{1}{{\sqrt {2\pi } }}\int_\alpha ^\infty {\exp } \left( { - \frac{{t^2}}{2}} \right)dt.$$

To generate the SAXS intensity from the two-phase pore structure we calculate the two-point probability function *P*_CC_(*r*) via

10 $$P_{{\mathrm{CC}}}\left( r \right) = \frac{1}{{2\pi }}\int_0^{g_Y(r)} {\frac{1}{{\sqrt {1 - t^2} }}} \exp \left( { - \frac{{\alpha ^2}}{{1 + t}}} \right)dt + \phi _{\mathrm{C}}^2.$$

The SAXS intensity of the empty carbon nanopore structure is generated by inserting *P*_CC_(*r*) in Eqs. (4) and (5) (Supplementary Fig. 9, Supplementary Table 2). Note that in previous papers the structure of the same activated carbon was modeled by intersecting two independently generated GRFs. Based on the plurigaussian random field approach applied in this work we do not use intersected GRF structures.

To model SAXS intensities and real space structures of the solid iodine phase (phase A) within the nanopores, we generate a second independent GRF *Z*(**x**) using the correlation function in Eqs. (7) and (8) with different input parameters *l*_*Z*_ and *d*_*Z*_ (Supplementary Fig. 10c). We set *d*_*Z*_ → ∞, i.e. the oscillation factor in Eq. (7) becomes 1 and $g_Z\left( r \right) = 1{\mathrm{/cosh}}(r{\mathrm{/}}l_Z)$. Phase A with the volume fraction *ϕ*_A_ within the pore structure is generated by cutting *Z*(**x**) and *Y*(**x**) according to Eq. (11) and the cut-offs visualized in Supplementary Fig. 10d–f.

11 $$\phi _{\mathrm{A}} = \iint_{(Y,Z){\it{\epsilon }}D_{\mathrm{A}}} {\frac{1}{{2\pi }}} {\mathrm{exp}}\left( { - \frac{{Y^2 + Z^2}}{2}} \right)dY\,dZ$$

The two-point correlation functions of phase A (and B) are calculated via

12 $$P_{{\mathrm{AA}}} = \int_{D_{\mathrm{A}}} {dY_1dZ_1} \int_{D_{\mathrm{A}}} {dY_2dZ_2 G_{g_Y\left( r \right)}} \left( {Y_1,Y_2} \right) G_{g_Z\left( r \right)}(Z_1,Z_2)$$

with *G*_*g*_(*Y*_1_, *Y*_2_) being the bivariate Gaussian distribution with mean 0, variance 1, and covariance *g*. Their calculation via Hermite polynomials is described in ref. ^45^. Depending on the angle *δ* and the A/B boundary line in Supplementary Fig. 10d–f the morphology of phase A will be different. Phase A will perfectly cover/wet the carbon phase in form of a thin film if *δ* → 0 (Supplementary Fig. 10d, g). In contrast, for an A/B boundary parallel to the Y-axis (*δ* → *π*/2) the structures of phase A(B) are statistically independent from phase C (Supplementary Fig. 10f, i). Inserting Eq. (12) into Eqs. (4) and (5) gives the corresponding scattering intensities (Supplementary Fig. 11). Sources of error and limitations of the plurigaussian model fit are discussed in Supplementary Note 1.

### Electrochemical characterization

Three-electrode Swagelok-type cells with a Ag/AgCl reference electrode were assembled for cyclic voltammetry (Supplementary Fig. 12), galvanostatic and potentiostatic charge/discharge measurements. The resulting rate capability, cycle life and the maximum amount of iodine that can be electrodeposited in the positive AC electrode (with self-discharge measurements) are shown in Fig. 4. We used an AC working electrode (diameter = 3 mm) with a mass of 0.3 mg, an AC counter-electrode (diameter = 10 mm) with a mass of 30 mg and about 250 µL electrolyte (three Whatman separators with 10 mm in diameter). This ensures that neither the total amount of I^−^ in the cell nor the double-layer capacitance of the counter-electrode is limiting the I_2_ uptake in the AC WE pores.

## Supplementary information

Supplementary Information

Peer Review File

Description of Additional Supplementary Files

Supplementary Movie 1

## Data Availability

The data that support the findings of this study are available from the corresponding authors on request.

## Code availability

The IgorPro (Wavemetrics) code used for in situ SAXS data analysis and stochastic modeling are available from the corresponding author C.P. on request.
