# Author Correction: Persistent and reversible solid iodine electrodeposition in nanoporous carbons

**DOI:** 10.1038/s41467-020-19720-x

**Published:** 2020-11-06

**Authors:** Christian Prehal, Harald Fitzek, Gerald Kothleitner, Volker Presser, Bernhard Gollas, Stefan A. Freunberger, Qamar Abbas

**Affiliations:** 1 Institute for Chemistry and Technology of Materials, Graz University of Technology, Stremayrgasse 9, 8010 Graz, Austria; 2 Graz Centre for Electron Microscopy, Steyrergasse 17, 8010 Graz, Austria; 3 Institute of Electron Microscopy and Nanoanalysis, NAWI Graz, Graz University of Technology, Steyrergasse 17, 8010 Graz, Austria; 4 INM - Leibniz Institute for New Materials, Campus D2 2, 66123 Saarbrücken, Germany; 5 Department of Materials Science and Engineering, Saarland University, Campus D2 2, 66123 Saarbrücken, Germany; 6 IST Austria (Institute of Science and Technology Austria), Am Campus 1, 3400 Klosterneuburg, Austria; 7 Institute of Chemistry and Technical Electrochemistry, Poznan University of Technology, Berdychowo 4, 60-965 Poznan, Poland; 8 Present Address: Department of Information Technology and Electrical Engineering, ETH Zürich, Gloriastrasse 35, 8092 Zürich, Switzerland

**Keywords:** Batteries, Batteries, Supercapacitors, Batteries, Electrochemistry

Correction to: *Nature Communications* 10.1038/s41467-020-18610-6, published online 24 September 2020.

This Article contains an error in part of the experimental procedure where some details were inadvertently omitted. The correct version is: “SAXS/WAXS data were recorded on a laboratory SAXS/WAXS facility (SAXSpoint 2.0, Anton Paar, Graz, Austria) using Cu-Kα radiation and a 2D areal detector (Eiger R 1M, Dectris, Baden, Switzerland) with a nominal sample-to-detector distance of 100 mm.”

The error has been corrected in the PDF or HTML versions of the Article.

