# Supplementary material for: Persistent and reversible solid iodine electrodeposition in nanoporous carbons
Source: Nat Commun. 2020 Sep 24;11:4838. doi: 10.1038/s41467-020-18610-6 (PMC7519142; doi:10.1038/s41467-020-18610-6)
Supplement: Supplementary file 1 — Supplementary Information [file 41467_2020_18610_MOESM1_ESM.pdf]

Supplementary Information

Persistent and reversible solid iodine electrodeposition in nanoporous carbons

Prehal *et al.*

Supplementary Figures

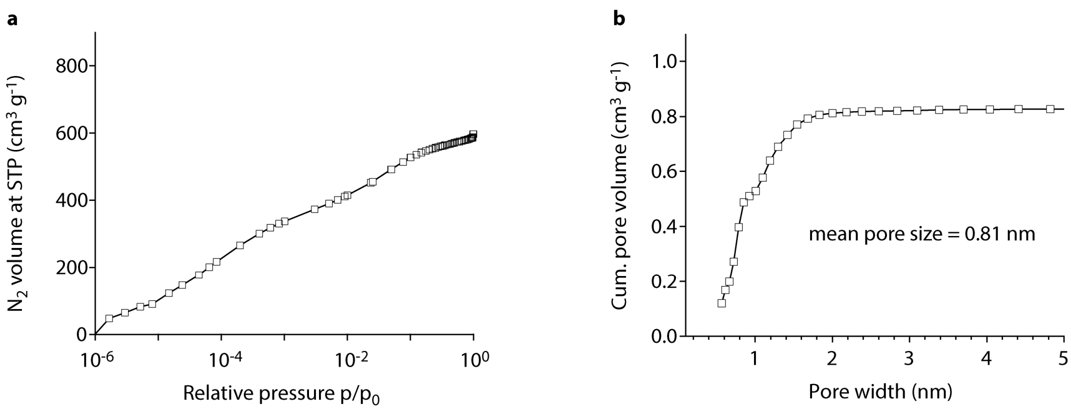

Supplementary Figure 1: (a), Nitrogen adsorption isotherm measured at -196 °C for the activated carbon powder. (b), Cumulative pore size distributions (PSDs) obtained from QSDFT assuming carbon pores with slit-shape.

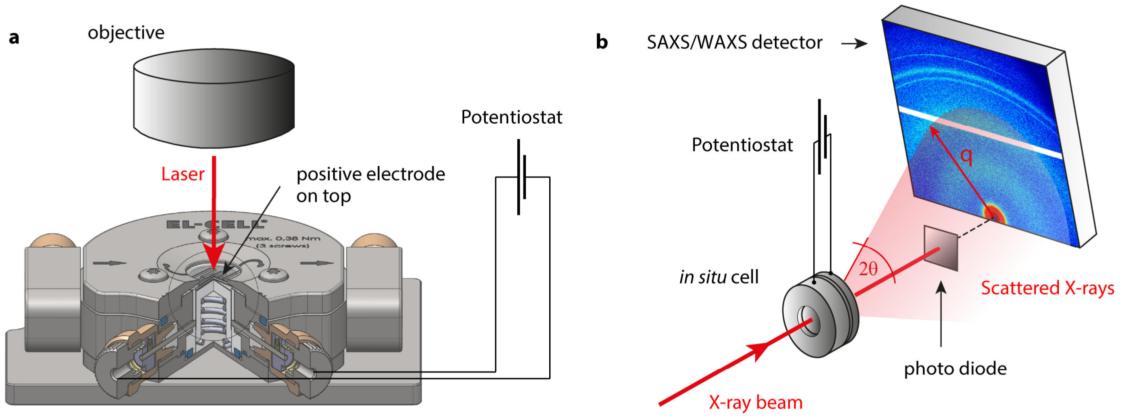

Supplementary Figure 2: Sketch of the experimental set-up of in situ Raman spectroscopy (a), and in situ small and wide-angle X-ray scattering (b).

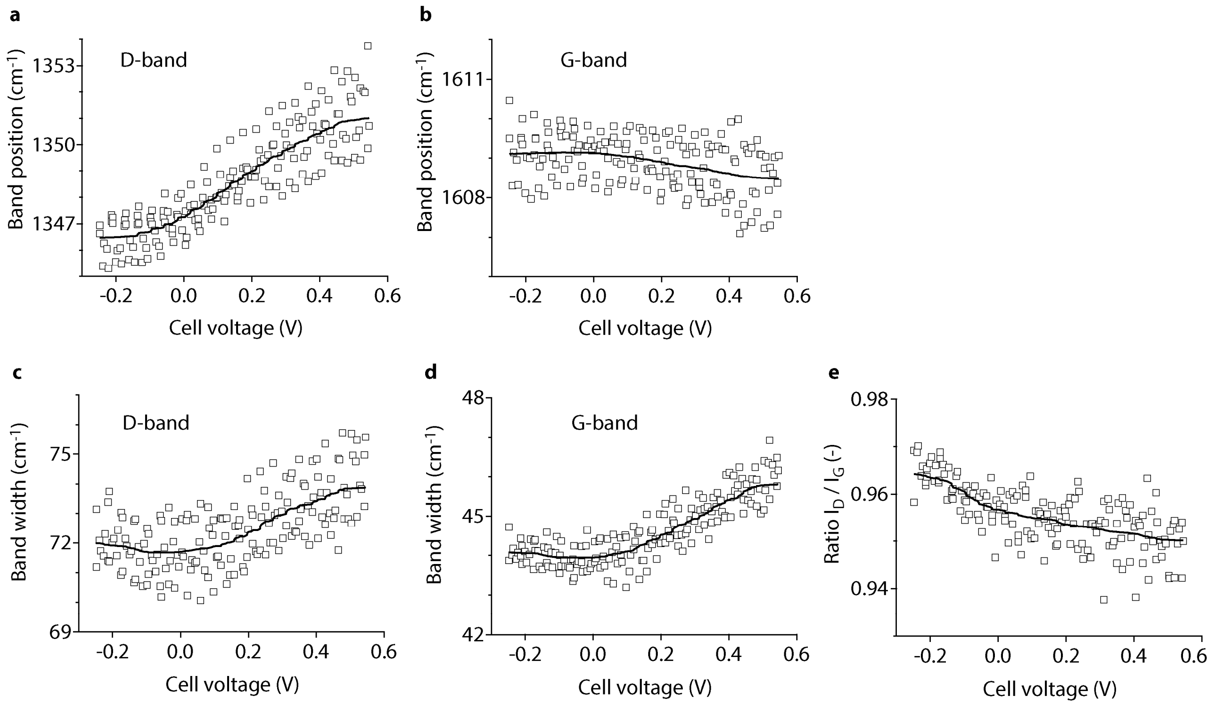

Supplementary Figure 3: D-band (a) and G-band (b) position as a function of the applied cell voltage. D-band (c) and G-band (d) width as a function of the applied cell voltage. (e), Ratio of D-band to G-band intensity as a function of the applied cell voltage. The solid red lines are generated by smoothing the experimental data (blue data points). Deconvolution of the G- and D- bands were done according to the procedure in Ref. 1.

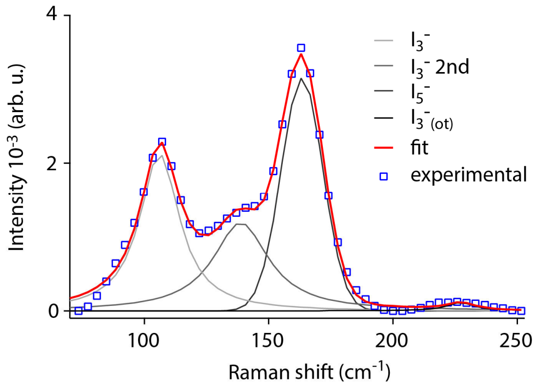

Supplementary Figure 4: Raman spectrum at 0.55 V cell voltage, taken from the in situ experiment shown in Fig. 1. Band deconvolution of the I_3^- and I_5^- bands were done using three Lorentz peaks (initial position 110 cm^{-1}, 143 cm^{-1}, and 224 cm^{-1}) and one Gaussian peak (initial position 165 cm^{-1}), according to Refs. 2,3.

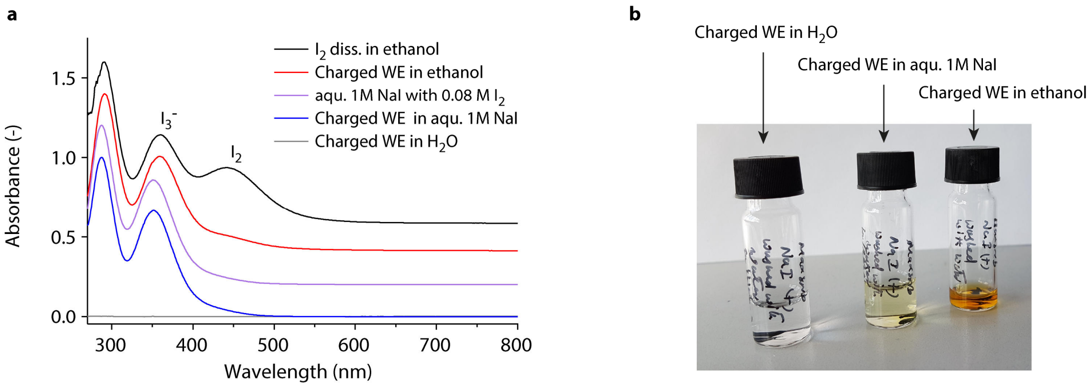

Supplementary Figure 5: (a), UV-vis absorption spectra of different (poly)iodide and iodine containing solutions. The ethanol solution in contact with the charged positive electrode (red curve) shows significant amounts of I_2 indicated by the shoulder around 460 nm. The significant amounts of I_3^- (peak around 370 nm) originate from a certain fraction of (poly-)iodides left in the pores of the charged positive electrode. Note that the absorption of I_3^- is much stronger than the absorption of I_2 (see black curve, where virtually no I_3^- should be present); the I_3^- and I_2 peak height ratio is hence not proportional to the ratio of their concentrations. (b) Photograph of charged positive electrodes (after positive polarization to 1.0 V cell voltage) immersed in de-ionized water (left), in an aqueous solution with 1 M NaI (center), and in ethanol (right).

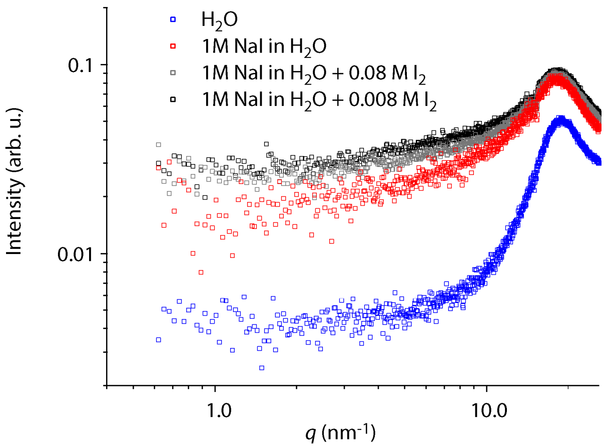

Supplementary Figure 6: Small and wide-angle X-ray scattering intensity (structure factors) of de-ionized H_2O, 1 M NaI in H_2O, 1 M NaI in H_2O + 0.008 M I_2 and 1 M NaI + 0.08 M I_2, recorded in quartz capillaries. Adding I_2 to the 1 M NaI electrolyte leads to the formation of polyiodides via chemical comproportionation.

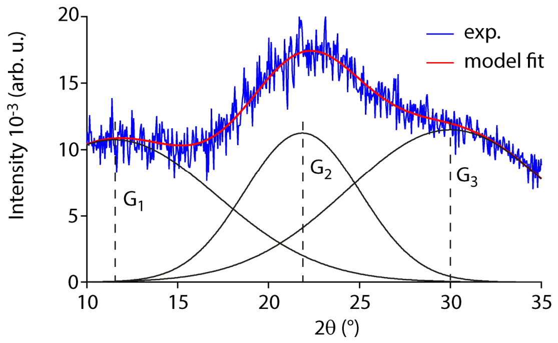

Supplementary Figure 7: Wide-angle X-ray scattering peak fit using three Gaussian peaks (black solid curves) with the parameters (x1=11.5°, sigma1=8°; x2=21.85°, sigma2=4.5°; x3=30.0°, sigma3=8°). G2 and G3 are attributed to the structure factor of solid iodine nanocrystals/nanoclusters. According to the Scherrer equation, their FWHM values correspond to a domain size (average coherence length) of 0.9 nm and 0.5 m, respectively.

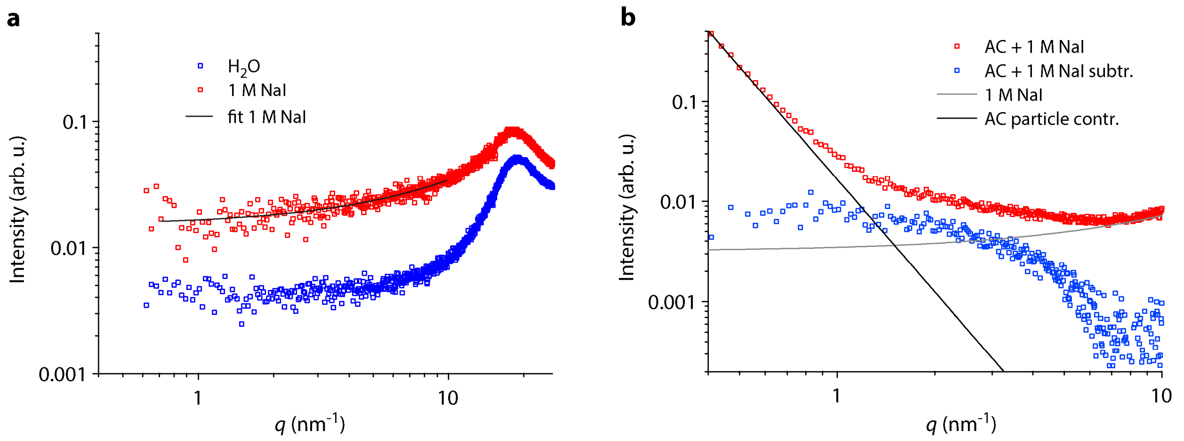

Supplementary Figure 8: (a), Electrolyte (1 M NaI in H_2O) and water structure factors recorded in quartz capillaries. The peak around 20 nm^{-1} corresponds to the H_2O structure factor peak. Adding (uncorrelated) Na^+ and I^- ions leads to an additive constant scattering intensity contribution with respect to the pure H_2O structure factor. The solid blue line is a polynomial fit of the third order. (b), To obtain the nanopore scattering contribution (blue data points) the electrolyte structure factor (solid grey line) and low q particle scattering contribution (solid black line) are subtracted from the scattering intensities recorded during the in situ SAXS/WAXS experiment (red data points), according to the procedure described in the experimental section.

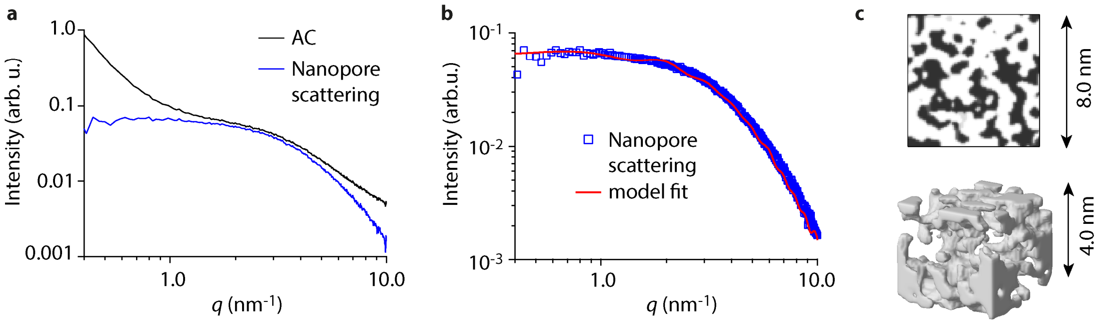

Supplementary Figure 9: 3D lattice model of carbon nanopores via Gaussian random fields and ex-situ SAXS. (a), The nanopore scattering intensity (blue solid line) is obtained by subtracting a constant background (determined via a standard Porod fit in the q-range 7 nm^{-1} < q < 9 nm^{-1}) and the particle scattering contribution (determined by a power-law fit for q < 1 nm^{-1}) from the SAXS intensity of the empty AC electrode (solid black line). (b), experimental nanopore scattering (blue data points) normalized by the integrated intensity (see experimental section), and modeled scattering intensity (solid red line) as a function scattering vector length q (model fit generation according to the description in the Section Methods). (c), Cross-section, and 3D cut-out of the resulting pore structure.

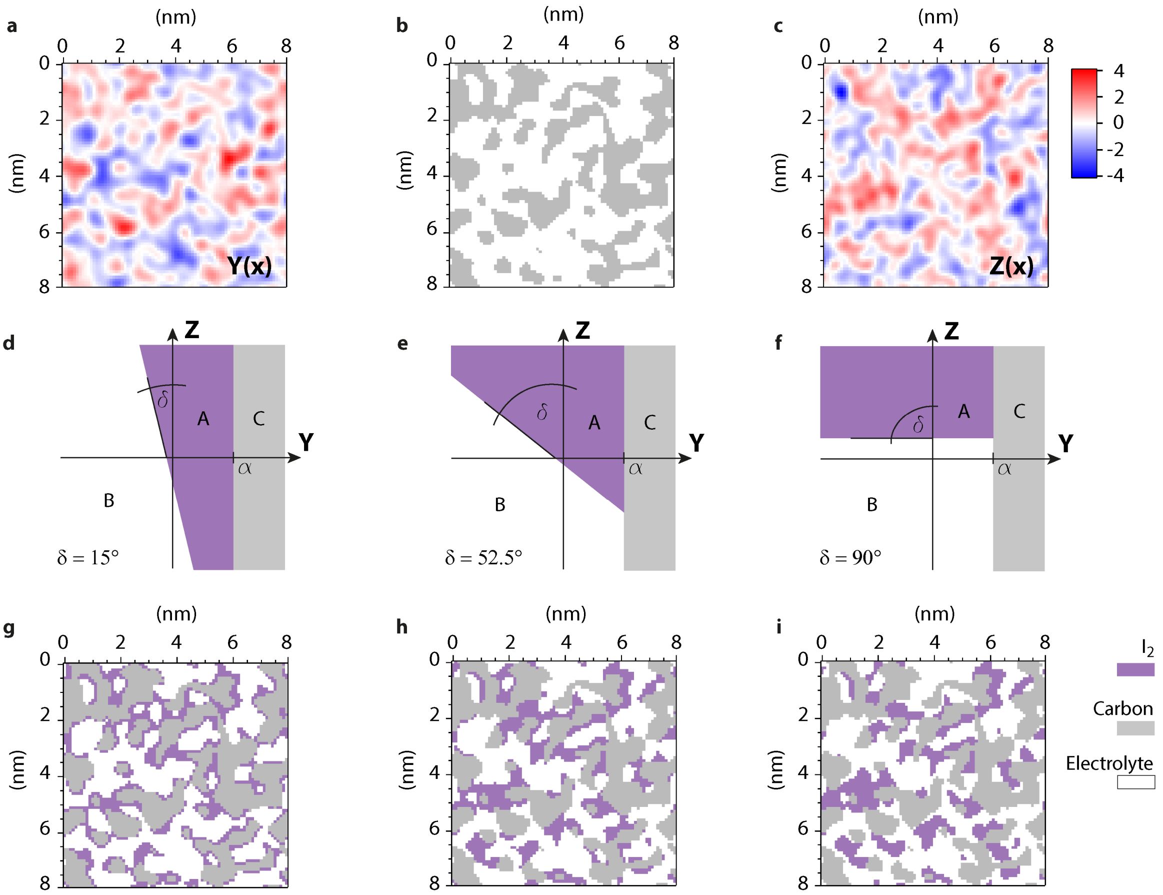

Supplementary Figure 10: (a), Cross-section of 3D Gaussian random field $Y(\boldsymbol{x})$ with the parameters $l_Y = 0.3\,nm, d_Y = 3.5\,nm, \alpha = 0.2$ used for generating the carbon pore model, as shown in (b). (c), cross-section of 3D Gaussian random field $Z(\boldsymbol{x})$ with the parameters: $l_Z = 0.3\,nm, d_Z = 3.5\,nm$ used for the iodine phase modeling. (d-e) Z-Y planes visualizing the threshold value dependencies of the two GRFs $Y(\boldsymbol{x})$ and $Z(\boldsymbol{x})$. The threshold value α to generate the carbon pore structure is indicated on the horizontal axis. The borderline between purple and white area determines whether phase A (I_2) shows a strong correlation (a, $\delta \rightarrow 0°$) or no correlation (c, $\delta \rightarrow 90°$) to phase C (carbon). The corresponding morphologies for $\delta = 15°$, $\delta = 52.5°$, and $\delta = 90°$ are shown in (g), (h) and (i), respectively. They range from an I_2 surface coating (d and g) to a completely uncorrelated iodine nanostructure (f and i).

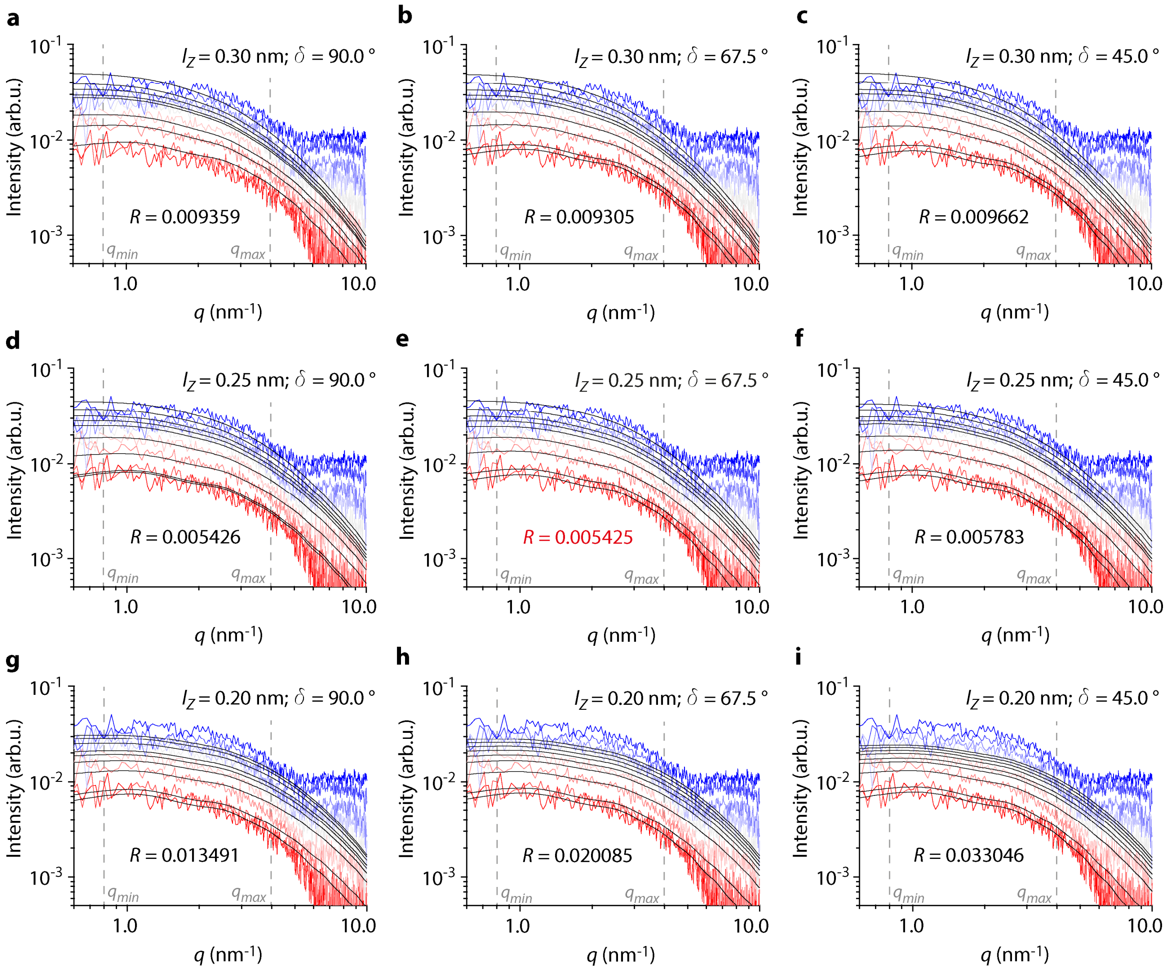

Supplementary Figure 11: Reduced experimental in situ small-angle scattering data during positive cell polarization (after subtraction of particle and electrolyte structure factor scattering contributions, as described in the experimental section and **Supplementary Figure 8**). I^- oxidation proceeds from red to solid blue lines. The modeled scattering intensities are shown as solid black lines, with the iodine pore occupancy used as the only fit parameter. In (a – i) the parameters l_Z and δ of the model scattering intensities are varied. The minimum value of the sum of the squared residuum values $R = \sum_i \sum_j \left(I_{i,exp}(q_j) - I_{i,mod}(q_j)\right)^2$ of all scattering curves i during positive cell polarization between $0.8\ nm^{-1} < q_j < 4.0\ nm^{-1}$ is taken as the solution of the model fit. The lowest R is found for $l_Z = 0.25\ nm$ and $\delta = 67.5\ °$ and the iodine pore occupancies ϕ_A given in **Figure 3c**.

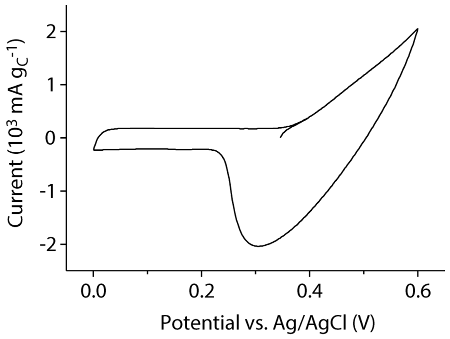

Supplementary Figure 12: (a), Cyclic voltammetry for a swagelok type three-electrode cell with 250 µL of 0.5 M NaI + 0.5 M $NaNO_3$ electrolyte and AC electrodes. The working electrode mass was 0.3 mg, the counter electrode mass was 30.0 mg.

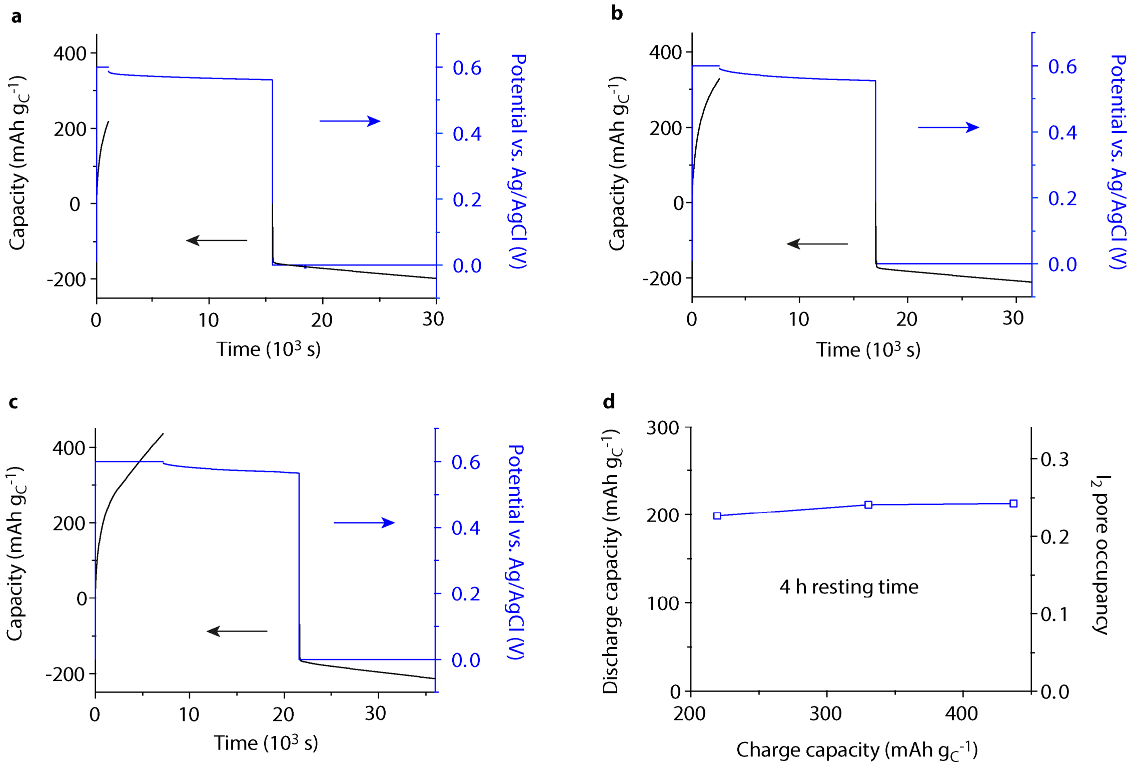

Supplementary Figure 13: Potentiostatic charge/discharge measurements with 4 h resting time in-between charging and discharging. The charging capacities correspond to (a) 219 mAh g_C^{-1}, (b) 330 mAh g_C^{-1}, and (c) 441 mAh g_C^{-1}. (d), the discharge capacities are similar already after 4 h of resting time. The right axis in (d) shows the theoretical iodine pore occupancy, assuming that all capacity originates from solid iodine and considering the porosity values given in **Supplementary** Table 1.

Supplementary Tables

Supplementary Table 1: Specific surface area (SSA) obtained from Brunauer-Emmett-Teller (BET) analysis, and quenched solid density-functional theory (QSDFT) for the AC powder. In addition, the mean pore size (d50) and the total specific pore volume determined with two different methods are given. The data are extracted from the N_2 adsorption data shown in **Figure S1**.

Carbons	BET SSA (m^2g^{-1})	QSDFT SSA (m^2g^{-1})	d_{50} (nm)	Total pore volume QSDFT (cm^3g^{-1})	Total pore volume at p/p0=0.95 (cm^3g^{-1})
AC	2098	1763	0.81	0.84	0.91

Supplementary Table 2: Fitting and input parameters for the 3D pore model generation using clipped Gaussian Random Fields[4]. The carbon volume fraction of the nanopores (intra-particular pores) is estimated via $\phi_C = 1 - V_{\text{spez., pore}}/(V_{\text{spez., pore}} + 1/\rho_{\text{Skelett}})$, where the carbon skeleton density was assumed with 1.9 g cm^{-3} and the specific pore volume taken from gas adsorption measurements (**Table S1**). The upper threshold value β is set to ∞ and relates to α via Equation S6. The fitting parameters l_Y and d_Y are obtained from fitting Equation S1 and Equation S2 to the normalized experimental SAXS curve in **Figure S9b**. The SAXS surface area of the GRF two-phase model is calculated by[5] $SSA_{\text{GRF}} = \frac{1}{\varphi_{\text{C}} * \rho_{\text{C.skel}}} \frac{2^{3/2}}{\pi} \exp\left(-\frac{\alpha^2}{2}\right) \frac{1}{l_{\text{C},Y}}$, where $\frac{1}{l_{\text{C},Y}{}^2} = \frac{1}{6} \int_0^\infty q^2 f_Y(q) dq$. The higher surface compared to the N_2 adsorption data is likely induced by narrow pores not accessible to the nitrogen molecule (<0.5 nm) during adsorption or density fluctuations[6] in the carbon skeleton.

Carbons	Carbon volume fraction ϕ_C	β	α	l_Y (nm)	d_Y (nm)	GRF SSA (m^2g^{-1})
AC	0.4	∞	0.275	0.27	3.2	3122

Supplementary Notes

Supplementary Note 1 | In situ SAXS model fit - limitations and sources of error

For the plurigaussian model fit, we used the iodine volume fraction ϕ_A, l_Z, and δ as fit parameters. Yet, to obtain the iodine volume fraction as a function of cell voltage, we fit each individual SAXS curve during charging by varying the volume fraction and keeping both l_Z and δ constant. The sum of the squared residuum values $\sum_i \sum_j \left(I_{i,exp}(q_j) - I_{i,mod}(q_j)\right)^2$ of all nine scattering curves i during positive cell polarization was then calculated using different l_Z and δ as visualized in Supplementary Fig. 11. The lowest value of this error sum was taken as the solution. This very coarse determination of l_Z and δ reflects the limited accuracy of the experimental nanopore scattering. Fitting l_Z and δ for each individual SAXS curve results in unphysical l_Z and δ changes during charging.

Systematic errors (induced by the delicate treatment of carbon, iodine/polyiodide, and electrolyte structure factor background) limit the meaningfulness of the nanopore scattering in situ data. Further, the correlation function used for the GRF Z(**x**), i.e. the model as such, does not not perfectly fit the real scattering data. Accordingly, differences in δ and l_z can only be detected with a certain accuracy. Supplementary Figure 11 shows modelled scattering intensities using the three different contact angles δ = 45°, 67.5°, and 90° and three different l_z = 0.20 nm, 0,25 nm, and 0.30 nm. Given the severe deviations between model and experiment and the systematic experimental errors, smaller δ and l_z variations make hardly any sense.

Within the (limited) accuracy we could not not detect severe structural differences during charge and discharge. Note the negligible differences in the SAXS intensiy shapes during charge and discharge in Supplementary Fig. 15a, b, below. Alternative correlation functions for the GRF Z(**x**), alternative carbons and improved data quality might help to improve the sensitivity of the plurigaussian model fit in future.

Supplementary Note 2 | Integrated Intensity analysis

The integrated intensity or invariant $\tilde{I}$ of the experimental SAXS intensity is numerically calculated by

$$\tilde{I} = \int_0^\infty q^2 I(q) dq \ . \tag{S1}$$

At high q the SAXS intensity requires a Porod extrapolation of the form $I(q) \propto q^{-4}$ (Fig. S14a,b). For a three-phase system consisting of phases A, B, C the integrated intensity can be written as[7]

$$\frac{\tilde{I}}{2\pi^2} = (\rho_A - \rho_B)(\rho_A - \rho_C)[\phi_A - \phi_A{}^2] + (\rho_B - \rho_A)(\rho_B - \rho_C)[\phi_B - \phi_B{}^2] + (\rho_C - \rho_A)(\rho_C - \rho_B)[\phi_C - \phi_C{}^2] \ . \tag{S2}$$

Here ρ_i is the electron density, and ϕ_i the volume fraction of phase i. Since carbon and micropore volume fractions (Supplementary Table 2) as well as electron densities are known, the iodine volume fraction can be calculated by inserting the experimental $\tilde{I}$ in equation S2.

The largest error with the integrated intensity analysis is induced by the delicate background subtraction and the corresponding Porod extrapolation at high q. At high potentials the polyiodide (I_3^-) correlations cause a peak around 8 nm^{-1}, which is difficult to separate from the nanopore scattering (see Supplementary Fig. 14a). The Kratky plot in Supplementary Fig. 14b indicates more clearly that the porod extrapolation is not ideal at higher cell voltages. This results in too high I_2 pore occupancies at high capacities (red curve, Supplementary Fig. 14c). Besides this deviation, the integrated intensity analysis confirms the quantities derived from the SAXS model fit and the electrochemical data.

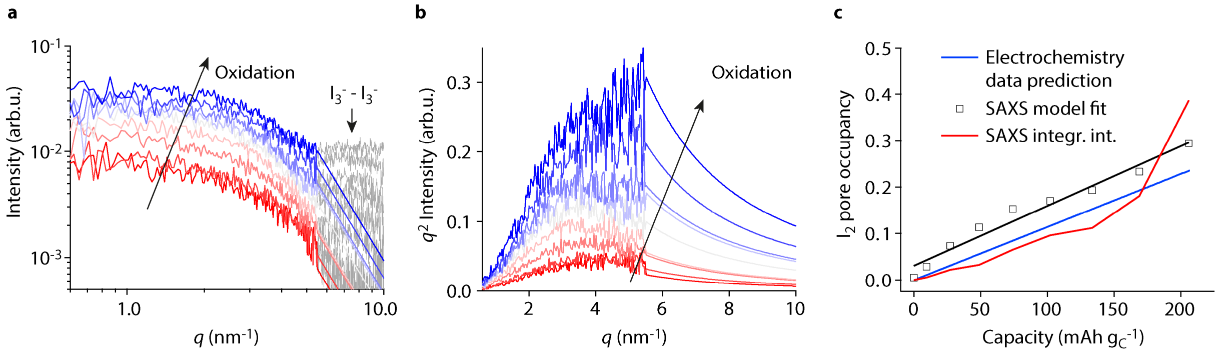

Supplementary Figure 14 | I_2 pore occupancy via integrated intensity: (a), nanopore scattering (Intensity vs. scattering vector length q) with Porod extrapolation at q > 5.5 nm^{-1} during oxidation/ with increasingly positive cell voltage. Since the polyiodide/iodine structure factor cannot be subtracted accurately, we have only subtracted the electrolyte structure at zero cell voltage and extrapolated the SAXS intensity at q > 5.5 nm^{-1} with $I(q) \propto q^{-4}$. (b), Kratky plot ($q^2 I(q)\ vs.\ q$) of the same curves. (c), I_2 pore occupancy as a function of capacity determined by the SAXS model fit, the estimation from electrochemical data (capacity) and the analysis of the SAXS integrated intensity. The latter is calculated by solving equation S2. The SAXS integrated intensity approach deviates at high capacities due to inaccurate Porod extrapolation.

Supplementary Note 3 | In situ SAXS potential dependency during charge and discharge

Supplementary Fig. 15a, b and the surface plot in Fig. 2d show that the iodine formation is nicely reversible. The SAXS and WAXS intensity fully drops to its initial values at zero cell voltage applied. To check for the reversibility and the time/voltage dependency we calculated the integrated intensity increase/decrease of the reduced experimental in situ SAXS data in the q-regime between $q_1 = 0.8$ nm^{-1} and $q_2 = 4.0$ nm^{-1} during two voltage cycles: $\tilde{I} = \int_{q_1}^{q_2} q^2 I(q) dq$. This implies that the absolute value of this parameter has no actual physical meaning, yet the relative change can accurately sense time and potential dependent changes of iodine formation. Supplementary Fig. 15d indicates a significant hysteresis of iodine formation/dissolution during charging/discharging. Since electrochemical charging and discharging is fast (as shown in Fig. 4), but iodide formation rather slow (Supplementary Fig. 15), electrochemical oxidation/reduction is (to a certain extend) decoupled from solid iodine formation. We believe that during I^- oxidation, I_2 is first dissolved, before it precipitates at sites where it is highly confined by the carbon. Whether the hysteresis is only a kinetic effect, (induced by fast cycling, iR drop), or incduced by an actual potential-dependency is investigated in the following.

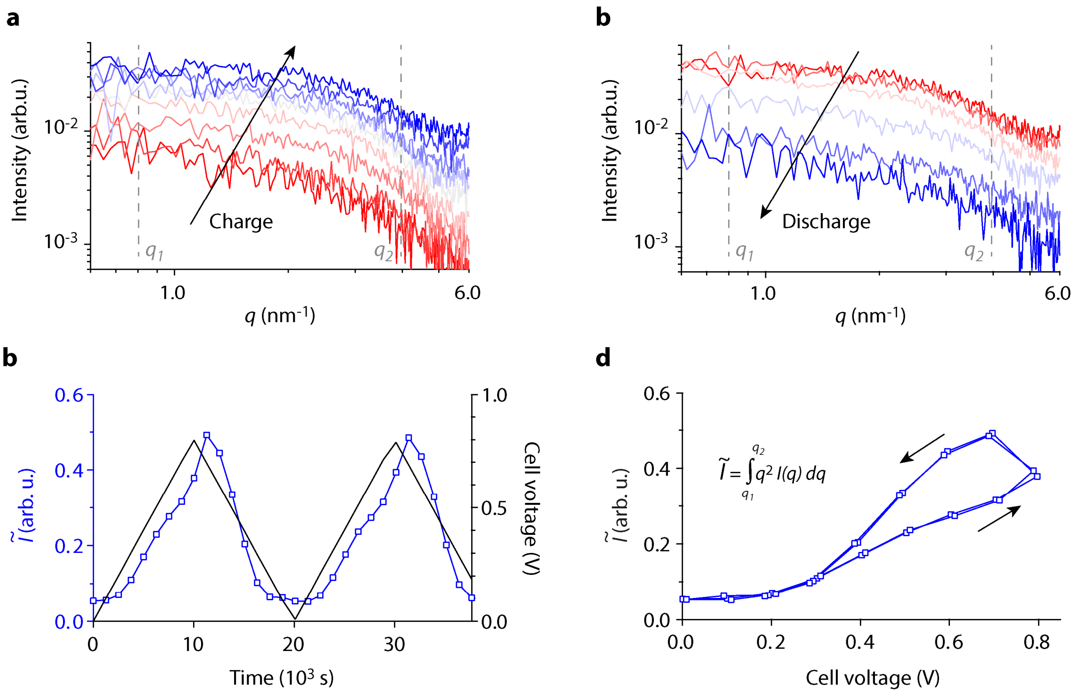

Supplementary Figure 15 | Reversibility of in situ SAXS data shown in Figs. 2 and 3: (a), Reduced experimental in situ SAXS data during positive cell polarization (after subtraction of particle and electrolyte structure factor scattering contributions, equivalent to Fig. 3a). (b) The equivalent for negative cell polarization. (c-d), Integrated intensity $\tilde{I}$ (with the integration borders $q_1 = 0.8$ nm^{-1} and $q_2 = 4.0$ nm^{-1}) of the reduced experimental in situ SAXS intensities as a function of time (a) and cell voltage (b). The black curve in (a) corresponds to the cell voltage versus time.

To carefully check for the potential dependency of iodine electrodeposition we carried out in situ SAXS measurements during potentiostatic charge/discharge. We mounted a silver wire as a reference electrode in the in situ SAXS cell and changed the working electrode potential step-wise (chronoamperometry)

Each potential was held constant for 30 min, until charging has basically stopped (Supplementary Fig. 15c). The SAXS/WAXS intensities increase during charge (Supplementary Fig. 15a) and reversibly decrease during discharge (Supplementary Fig. 15b). In Supplementary Fig. 15d, the integrated intensity value of the reduced experimental in situ SAXS data (nanopore scattering, equivalent to what is shown in Fig. 3a or Supplementary Fig. 11) at the end of each 30 min potential step is given as a function of the WE potential. The significant hysteresis points at an intrinsic overpotential necessary to dissolve iodine during discharge and the increased stability of I_2 in nanoporous confinement due to carbon - iodine interactions. From a physico-chemical point of view, this might be explained by interface energies between carbon, iodide and electrolyte. Given the complexity of the system, the interpretation of the hysteresis needs to be treated with caution.

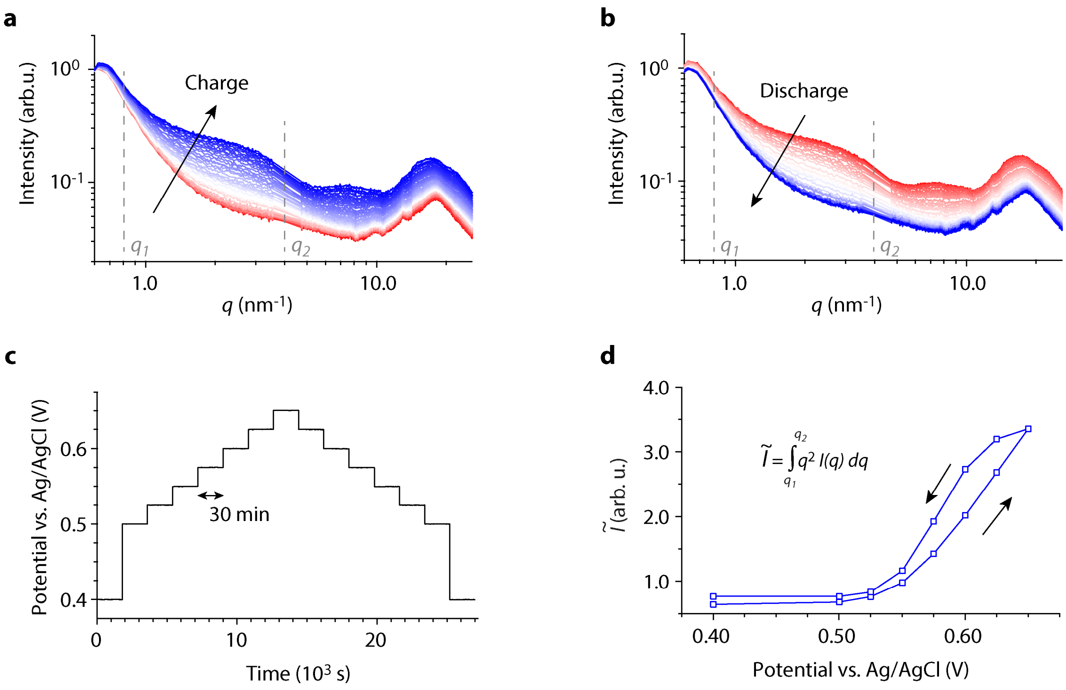

Supplementary Figure 16 | SAXS potential dependency during positive and negative cell polarization: (a), scattering intensity versus scattering vector length q during potentiostatic charging in several steps from 0.40 V to 0.65 V vs. Ag/AgCl, as shown in (c). (b), scattering intensity versus scattering vector length q during potentiostatic discharging in several steps from 0.65 V to 0.40 V vs. Ag/AgCl, as shown in (c). (c), Applied WE potential vs. Ag/AgCl as a function time. (d), Integrated intensity $\tilde{I}$ of reduced experimental in situ SAXS intensities (after subtraction of particle and electrolyte structure factor scattering contributions, equivalent to Fig. 3a and Supplementary Fig. 11) as a function of the WE potential. The integrated intensity is calculated for the q-regime between q_1 = 0.8 nm^{-1} and q_2 = 4.0 nm^{-1} at the end of each 30 min potential step shown in (c). The integrated intensity shows a clear hysteresis, indicating that I_2 dissolution requires some overpotential during discharge.

Supplementary References

1 Ferrari, A. C. & Robertson, J. Interpretation of Raman spectra of disordered and amorphous carbon. *Phys. Rev. B* **61**, 14095-14107 (2000).

2 Milne, J. A Raman spectroscopic study of the effect of ion-pairing on the structure of the triiodide and tribromide ions. *Spectrochim. Acta A* **48**, 533-542 (1992).

3 Sharp, S. B. & Gellene, G. I. Ab Initio Calculations of the Ground Electronic States of Polyiodide Anions. *J. Phys. Chem. A* **101**, 2192-2197 (1997).

4 Prehal, C. *et al.* A carbon nanopore model to quantify structure and kinetics of ion electrosorption with in situ small angle X-ray scattering. *Phys. Chem. Chem. Phys.* **19**, 15549 (2017).

5 Gommes, C. J. Stochastic models of disordered mesoporous materials for small-angle scattering analysis and more. *Microporous Mesoporous Mater.* **257**, 62-78 (2018).

6 Ruland, W. Small-angle scattering of two-phase systems: determination and significance of systematic deviations from Porod's law. *J. Appl. Crystallogr.* **4**, 70-73 (1971).

7 Gommes, C. Three-dimensional reconstruction of liquid phases in disordered mesopores using in situ small-angle scattering. *J. Appl. Crystallogr.* **46**, 493-504 (2013).
