# Supplementary material for: Persistent and reversible solid iodine electrodeposition in nanoporous carbons
Source: Nat Commun. 2020 Sep 24;11:4838. doi: 10.1038/s41467-020-18610-6 (PMC7519142; doi:10.1038/s41467-020-18610-6)
Supplement: Supplementary file 3 — Description of Additional Supplementary Files [file 41467_2020_18610_MOESM3_ESM.pdf]

Description of Additional Supplementary Files

File name: Supplementary Movie 1

Description: Three-dimensional 4 nm x 4 nm x 4 nm cut-out of the activated carbon (grey) filled with 30% iodine (violet). The structures are modelled using plurigaussian random fields.
