# Supplementary material for: Persistent and reversible solid iodine electrodeposition in nanoporous carbons
Source: Nat Commun. 2020 Sep 24;11:4838. doi: 10.1038/s41467-020-18610-6 (PMC7519142; doi:10.1038/s41467-020-18610-6)
Supplement: Supplementary file 2 — Peer Review File [file 41467_2020_18610_MOESM2_ESM.pdf]

Reviewer #1 (Remarks to the Author):

This manuscript shows a very good example: from mechanical understanding to guiding the real system. The authors report on the iodine electrochemistry mechanism in nanoporous carbon, which is very well performed with in situ Raman and in situ ASXS/WAXS. They elegantly explained the increased capacity densities and reduced self-discharge. Equipped with the mechanistic picture, they also derive handles, such as support electrolyte and the concentration of iodide, towards elegant iodine electrochemistry. In short, the study provides very significant new insights into the iodide electrochemical mechanism and governing factor (electrolyte and electrode) to complete better electrochemical performance. In addition, the work is also useful for the study of other battery systems (Li-S batteries, Zn-I2 batteries and so on), and will certainly receive widespread attention. Therefore, I am very glad to see this manuscript published in Nature Communication as soon as possible. Another topic of interest to reviewers is how iodine electrochemistry works in organic solvents. The authors should comment on it in the introduction.

Reviewer #2 (Remarks to the Author):

The manuscript “Persistent and Reversible Solid Iodine Electrodeposition in Nanoporous Carbons” by Prehal et al, investigates the nanometer-scale physicochemical phenomena involved in the loading of iodine-based supercapacitors. The study is based on in-situ Raman spectroscopy, together with (independent) in-situ small- and wide-angle x-ray scattering, measured during electrochemical loading/unloading cycles.

The central claim of the manuscript is that the oxidation of iodide at the positive electrode results in stable iodine nanocrystals in the pores of the activated carbon that makes up the electrode. The experiments are carefully conducted, the results are novel, interesting and prone to modify the thinking in the field of supercapacitors in general.

The two main points I raise hereunder concern (i) a question of small-angle scattering data analysis, which should be clarified, and (ii) some relevant physicochemical considerations which are not touched on in the manuscript.

The very existence of solid iodine is supported qualitatively by the reversible appearance of a broad feature in the wide-angle scattering (WAXS) patterns during oxidation (Fig. 2g). WAXS cannot be used to ascertain whether that crystal accounts for a significant fraction of the capacity, or if it is merely a trace. Small-angle scattering (SAXS) is the only technique in the manuscript that can be used to quantitatively estimate the amount of solid iodine that is actually present in the pore. In that respect, a central figure in the manuscript is Fig. 3c. It is, however, not entirely clear how the pore occupancy was obtained from the SAXS.

A relatively straightforward approach would consist in analyzing the integrated SAXS intensity. This would provide one with an estimate of the iodine fraction that is independent of any model (plurigaussian or other). The authors mention measuring the integrated intensity, but they seem to use it to normalize the scattering patterns. If that is the case, it is unclear how volume fractions can be obtained at all from the SAXS. Moreover, the results should depend critically on the electron densities of the phases, but the used values are found neither in the manuscript nor in the Supporting Information.

From a physicochemical point of view, it would also be interesting to understand why iodine confined in carbon would tend to form stable crystals at all. The sheer fact that the WAXS of iodine is shifted to

lower q compared to the bulk (Fig. 2g) hints at tensile stresses. Such negative pressure would result in reduced chemical potential, and increased stability. Perhaps this can be explained in terms of the Hamaker constants of the various phases. Incidentally the strong interactions between carbon and iodine, which have to be invoked to explain the crystal stability, would necessarily lead to hysteresis. One would expect the crystals to be even more stable for decreasing (positive) potentials than for increasing potentials. This is not discussed at all in the manuscript. In that respect, it would be interesting to see the equivalent of Figs 2f and 2g for decreasing potentials too. It would also be particularly interesting to see if the plurigaussian model can detect differences in contact angles (delta) for increasing and decreasing potentials.

Please find hereunder other relatively minor points.

• How critical are the other parameters of the plurigaussian model for the determination of the iodine volume fraction (Fig. S11)? What does Fig. 3c look like if other values are used for lZ and delta?

• How does the surface area determined from nitrogen physisorption compare to the one obtained from SAXS?

• Insets of Fig. 2e and 2f: Wouldn't one expect a contrast-matching effect, whereby the SAXS signal would initially decrease as solid iodine would bring the electron density of the pore-filling material closer to that of carbon?

• Caption of Fig S10. What is the relevance of parameter alpha for field Z?

• Page 5, lines 4-6: Doesn't the attenuation depend on the total amount of iodide, iodine, etc. over the optical path of the x-ray, independently of where these species are positioned?

• In many instances through the paper, the unit of the lengths that enter the plurigaussian model is not specified (e.g. Table S2, Captions of Figs. S10 and S11). Please correct that.

• Page 15, Eq. 9: I believe alpha and beta have been swapped in the integration limits.

• Page 15, Eq. 10. Unless I am mistaken, parameter beta is set to infinity for all the models used in the paper. If that is the case, I suggest not mentioning beta at all, and showing only the (simpler) equations in the limit of beta equal infinity.

With kind regards,

Reviewer #3 (Remarks to the Author):

This work proposes the reaction mechanism during iodine deposition in microporous carbon electrode and suggests that reducing the concentration of I- in the electrolyte assists the formation of more I2 and hence mitigates the self-discharge of carbon in the iodide based electrolyte. The mechanism proposed in this study is interesting and the characterization supports well the mechanism proposed. However, there are several major concerns:
1. The paper suggests that the strategy proposed in this study can also be applied to Li-S battery. It is true that the capacity degrades in the Li-S battery because of the loss of polysulfide during the charge/discharge process. But it is difficult to find how the strategy in this study could help the Li-S battery because of the difference between the charging mechanism of Li-S and iodine battery. The iodine formed in the iodine battery comes from the electrolyte, and will turn back to the iodides during discharge. Controlling the concentration of I- in the electrolyte may assist the I2 deposition. In Li-S battery, the major strategy is to keep the S species within the electrode. The Li+ intercalation, instead

of S species, contributes to the charge storage in the Li-S battery, which is different from I2 battery,

2. The iodine battery works in the way that the ion in the liquid phase transforms to solid-phase I2 in the confined space. The improved I2 pore occupancy improves the capacity, but at the same time, it is at the cost of the rate capability. When more ions transform into the solid phase, the pore will be more blocked and slower ion diffusion can be expected.

3. It is important to take the amount of electrolyte used in each cell into consideration. As the I- concentration in the electrolyte will decrease during the process, it is not a sustainable process. Or, it is suggested to quantify the ion centration during charging. Also, it is better to show the Coulombic efficiency of the electrode.

4. The advantage of the iodine battery is unclear. There is only one electron transfer per I- if all I- transfer to I2, and the capacity of the device depend highly on the pore volume of the AC. The voltage window is very narrow, even narrower than the electrical double layer capacitor, meaning the energy density of the device will be limited. Moreover, the rate capability and cycling stability are not reported here. It is important to claim the advantage of this type of battery.

5. The battery shows a higher capacity in 0.5 M: 0.5 M NaI: NaNO3 than that in 1M NaI. The improved capacity could be due to complete transformation from I- to I2. But it seems that the self-discharge becomes worse as the capacity drops faster in 0.5 M: 0.5 M NaI: NaNO3 electrolyte.

Response to reviewers on the paper entitled

"Persistent and Reversible Solid Iodine Electrodeposition in Nanoporous Carbons"

Manuscript: NCOMMS-20-13228

We thank the Reviewers for their helpful comments to which we respond below point by point. The Reviewers' comments are reproduced in italics. In the manuscript and supporting information we highlighted the changes in yellow. The comments helped us greatly to improve the manuscript and we are confident that we have satisfyingly responded to all of them.

Reviewer #1:

1.1. This manuscript shows a very good example: from mechanical understanding to guiding the real system. The authors report on the iodine electrochemistry mechanism in nanoporous carbon, which is very well performed with in situ Raman and in situ ASXS/WAXS. They elegantly explained the increased capacity densities and reduced self-discharge. Equipped with the mechanistic picture, they also derive handles, such as support electrolyte and the concentration of iodide, towards elegant iodine electrochemistry. In short, the study provides very significant new insights into the iodide electrochemical mechanism and governing factor (electrolyte and electrode) to complete better electrochemical performance. In addition, the work is also useful for the study of other battery systems (Li-S batteries, Zn-I2 batteries and so on), and will certainly receive widespread attention. Therefore, I am very glad to see this manuscript published in Nature Communication as soon as possible. Another topic of interest to reviewers is how iodine electrochemistry works in organic solvents. The authors should comment on it in the introduction.

We thank Rev. 1 for this very positive feedback. We have added the following statement related to organic electrolytes to the introduction:

"Works on carbon - iodine battery cathodes[1, 2] suggest that the physico-chemical mechanism during iodide/iodine oxidation/reduction is similar in both organic and aqueous electrolytes."

[1] *Y. L. Wang et al., Energy Environ. Sci. 4, 10 (2011): 3947-3950.*
[2] *Q. Zhao et al., Nano Letters 15, 9 (2015): 5982-5987.*

Reviewer #2:

The manuscript "Persistent and Reversible Solid Iodine Electrodeposition in Nanoporous Carbons" by Prehal et al, investigates the nanometer-scale physicochemical phenomena involved in the loading of iodine-based supercapacitors. The study is based on in-situ Raman spectroscopy, together with (independent) in-situ small- and wide-angle x-ray scattering, measured during electrochemical loading/unloading cycles. The central claim of the manuscript is that the oxidation of iodide at the positive electrode results in stable iodine nanocrystals in the pores of the activated carbon that makes up the electrode. The experiments are carefully conducted, the results are novel, interesting and prone to modify the thinking in the field of supercapacitors in general.

We thank reviewer 2 for the positive feedback and very constructive comments. We have added new in situ SAXS data, deepened discussions regarding the SAXS data analysis/interpretation and added the analysis of the SAXS integrated intensity to the SI.

The two main points I raise hereunder concern (i) a question of small-angle scattering data analysis, which should be clarified, and (ii) some relevant physicochemical considerations which are not touched on in the manuscript.

2.1. The very existence of solid iodine is supported qualitatively by the reversible appearance of a broad feature in the wide-angle scattering (WAXS) patterns during oxidation (Fig. 2g). WAXS cannot be used to ascertain whether that crystal accounts for a significant fraction of the capacity, or if it is merely a trace. Small-angle scattering (SAXS) is the only technique in the manuscript that can be used to quantitatively estimate the amount of solid iodine that is actually present in the pore. In that respect, a central figure in the manuscript is Fig. 3c. It is, however, not entirely clear how the pore occupancy was obtained from the SAXS.

A relatively straightforward approach would consist in analyzing the integrated SAXS intensity. This would provide one with an estimate of the iodine fraction that is independent of any model (plurigaussian or other). The authors mention measuring the integrated intensity, but they seem to use it to normalize the scattering patterns. If that is the case, it is unclear how volume fractions can be obtained at all from the SAXS. Moreover, the results should depend critically on the electron densities of the phases, but the used values are found neither in the manuscript nor in the Supporting Information.

The biggest issue with using the integrated intensity is the delicate background subtraction and the corresponding Porod extrapolation at high q. At high potential, the polyiodide (I_3^-) correlations cause a peak around 8 nm^{-1}, which is difficult to separate from the nanopore scattering (see Fig. 2f and Fig. R2.1a). Nevertheless, we have now added an estimation of the I_2 pore occupancy evolution based on the integrated intensity analysis to Supplementary Note 2.

Initially, we have only normalized the SAXS intensity of the *empty* carbon electrode by the integrated intensity. The in situ SAXS data have not been normalized. Instead, all modelled SAXS intensities have been multiplied by the constant K in equation 4, which was determined by dividing the integrated intensity of the nanopore scattering intensity at zero cell voltage by the integrated intensity of the corresponding modelled SAXS intensity with zero Iodine fraction. This has been clarified in the methods section. SAXS intensities are now treated consistently both for the plurigaussian model fit of the in situ SAXS data (Supplementary Fig. 11) and the GRF model fit of the empty carbon electrode structure (Supplementary Fig. 9).

Since the background subtraction makes the integrated intensity analysis inaccurate at high depths of charge, we have estimated the iodine pore occupancy by the model fits shown in Supplementary Fig. 11. The exact shape and height of intensities in Fig. 3a depend sensitively on the procedure to separate the nanopore scattering and electrolyte/iodide structure factors. The experimental in situ SAXS data were corrected by subtracting an electrolyte structure factor contribution (which is assumed to remain constant during cycling), determined from a Porod fit to the SAXS intensity at zero cell voltage. The structure factor contribution stemming from polyiodide interactions or the iodine atomic structure is not subtracted. For that reason, the SAXS data were fitted by the plurigaussian model in the limited q-regime $0.8 < q < 4.0$ nm^{-1} (Supplementary Fig. 11).

We used the iodine pore occupancy, l_Z, and δ as fit parameters. Yet, to obtain the pore occupancy as a function of cell voltage, we fit each individual SAXS curve during charging by varying the pore occupancy and keeping both l_Z and δ constant. The sum of the squared residuum values $\sum_i \sum_j \left(I_{i,exp}(q_j) - I_{i,mod}(q_j)\right)^2$ of all nine scattering curves i during positive cell polarization is then calculated using different l_Z and δ as visualized in Supplementary Fig. 11. The lowest value of this error sum is taken as the solution. This very coarse determination of l_Z and δ reflects the limited accuracy of the experimental nanopore scattering. Fitting l_Z and δ for each individual SAXS curve results in unphysical l_Z and δ changes during charging.

The scattering length densities of iodine, carbon, and electrolyte are assumed with $3.49 \cdot 10^{11}$ cm^{-2}, $1.61 \cdot 10^{11}$ cm^{-2}, and $1.01 \cdot 10^{11}$ cm^{-2}, respectively. This corresponds to a mass density of 1.9 g·cm^{-3} for the carbon skeleton, following previous works[1], and 4.93 g·cm^{-3} for iodine, corresponding to the iodine crystal density. The scattering length density of the electrolyte corresponds to a concentration of 1 M NaI in the aqueous solvent. These numbers are now mentioned in the figure caption of Fig. 2 and the methods section.

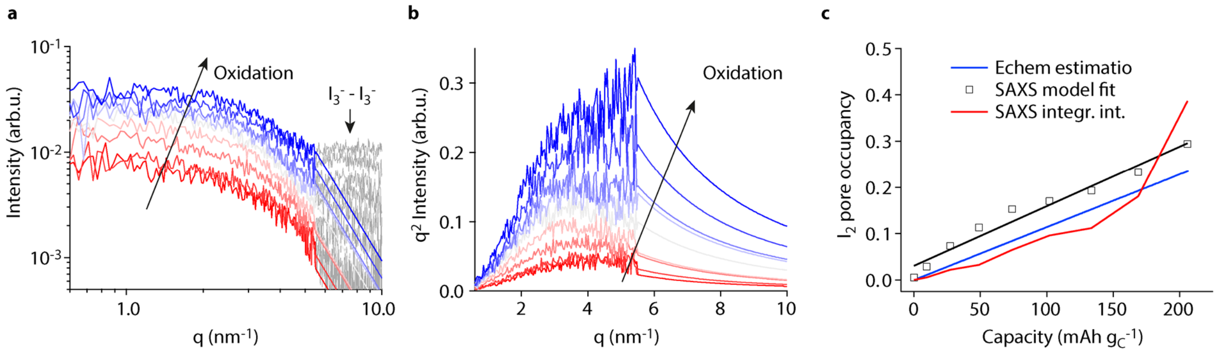

Figure R2.1 (new Supplementary Fig. 14) | I_2 pore occupancy via integrated intensity: (a), nanopore scattering (Intensity vs. scattering vector length q) with Porod extrapolation at q > 5.5 nm^{-1} during oxidation/ with increasingly positive cell voltage. Since the polyiodide/iodine structure factor cannot be subtracted accurately, we have only subtracted the electrolyte structure at zero cell voltage and extrapolated the SAXS intensity at $q > 5.5$ nm^{-1} with $I(q) \propto q^{-4}$. (b), Kratky plot ($q^2 I(q)\ vs.\ q$) of the same curves. (c), I_2 pore occupancy as a function of capacity determined by the SAXS model fit, the estimation from electrochemical data (capacity), and the analysis of the SAXS integrated intensity. The latter is calculated by solving equation S2 in Supplementary Note 1. The SAXS integrated intensity approach deviates at high capacities due to inaccurate Porod extrapolation.

[1] *C. Prehal et al., Carbon 152 (2019): 416-423.*

2.2. From a physicochemical point of view, it would also be interesting to understand why iodine confined in carbon would tend to form stable crystals at all. The sheer fact that the WAXS of iodine is shifted to lower q compared to the bulk (Fig. 2g) hints at tensile stresses. Such negative pressure would result in reduced chemical potential, and increased stability. Perhaps this can be explained in terms of the Hamaker constants of the various phases. Incidentally the strong interactions between carbon and iodine, which have to be invoked to explain the crystal stability, would necessarily lead to hysteresis. One would expect the crystals to be even more stable for decreasing (positive) potentials than for increasing potentials. This is not discussed at all in the manuscript. In that respect, it would be interesting to see the equivalent of Figs 2f and 2g for decreasing potentials too. It would also be particularly interesting to see if the plurigaussian model can detect differences in contact angles (delta) for increasing and decreasing potentials.

In general, more systematic studies with different carbons and an increased sensitivity of the plurigaussian model fit (e.g. by using other correlation functions) are necessary to conclusively answer all of these questions.

Fig. R2.2a, b and the surface plot in Fig. 2d show that the iodine formation is nicely reversible. The SAXS and WAXS intensity fully drops to its initial values at zero cell voltage applied. To check for the reversibility and the time/voltage dependency we calculated the integrated intensity increase/decrease of the nanopore scattering (see e.g. Fig. 3a) in the q-regime between q_1 = 0.8 nm^{-1} and q_2 = 4.0 nm^{-1} during two voltage cycles. The q-regime was limited due to the difficult background subtraction, as discussed above. This implies that the absolute value of this parameter has no actual physical meaning, yet the relative change should accurately sense time and potential dependent changes of iodine formation. Fig. R2.2d indicates a significant hysteresis of iodine formation/dissolution during charging/discharging. As mentioned by the reviewer, the hysteresis might be a good indicator for the increased stability of iodine in the nanopore confinement. Since electrochemical charging and discharging is fast (as shown in Fig. 4), but iodide formation rather slow (Supplementary Fig. 15), electrochemical oxidation/reduction is (to a certain extend) decoupled from solid iodine formation. This suggests that during I^- oxidation, I_2 is first dissolved, before it precipitates at sites where it is highly confined by the carbon.

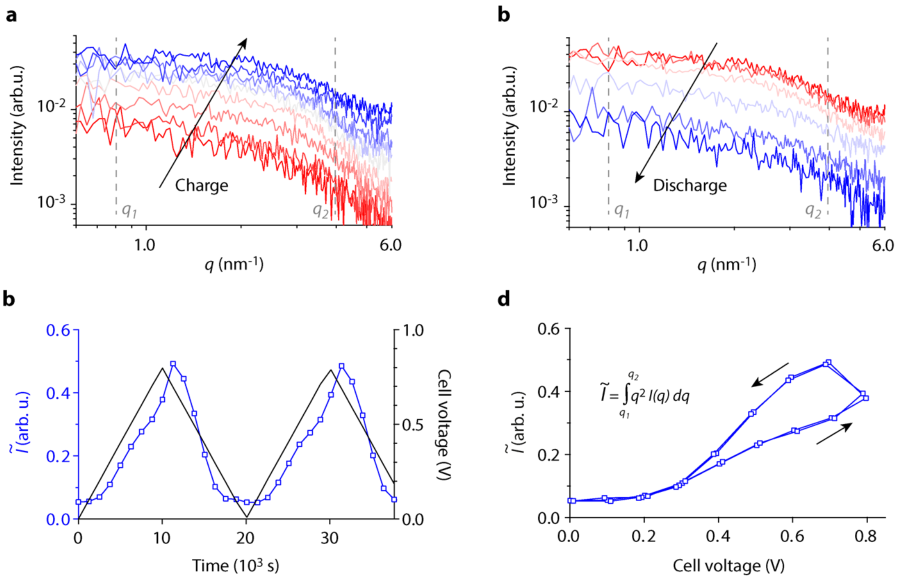

Figure R2.2 (new Supplementary Fig. 15) | Reversibility of in situ SAXS data shown in Fig.2 and 3: (a), Reduced experimental in situ SAXS data during positive cell polarization (after subtraction of particle and electrolyte structure factor scattering contributions, equivalent to Fig. 3a). (b) The equivalent for negative cell polarization. (c-d), Integrated intensity $\tilde{I}$ (with the integration borders q_1 = 0.8 nm^{-1} and q_2 = 4.0 nm^{-1}) of the reduced experimental in situ SAXS intensities as a function of time (a) and cell voltage (b). The black curve in (a9 corresponds to the cell voltage versus time.

Whether the hysteresis is only a kinetic effect, (induced by fast cycling, iR drop), or due to an actual potential-dependency is investigated in the following. We have carried out new in situ SAXS measurements, where we mounted a silver wire as a reference electrode in a new in situ SAXS cell and changed the working electrode potential step-wise (chronoamperometry). Each potential was held at a constant value for 30 min, until electrochemical charging/discharging has finished (Fig. R2.3c). The SAXS/WAXS intensities increase during charge (Fig. R2.3a) and decrease (fully reversible) during discharge (Fig. R2.3b). In Fig. 2d, the integrated intensity value of the reduced experimental in situ SAXS data (nanopore scattering, equivalent to what is shown in Fig. 3a or Supplementary Fig. 11) at the end of each 30 min potential step is given as a function of the WE potential. The significant hysteresis points at an intrinsic overpotential necessary to dissolve iodine during discharge and the increased stability of I_2 in nanoporous confinement due to carbon - iodine interactions. This may be explained via interfacial energies (or Hamaker constants) of the three phases, as suggested by the reviewer. Given the complexity of the system these interpretations have to be treated with caution. Making more quantitative statements in that regard requires further investigations and theoretical considerations in future.

This is now discussed in Supplementary Note 3 and mentioned in main part of the text.

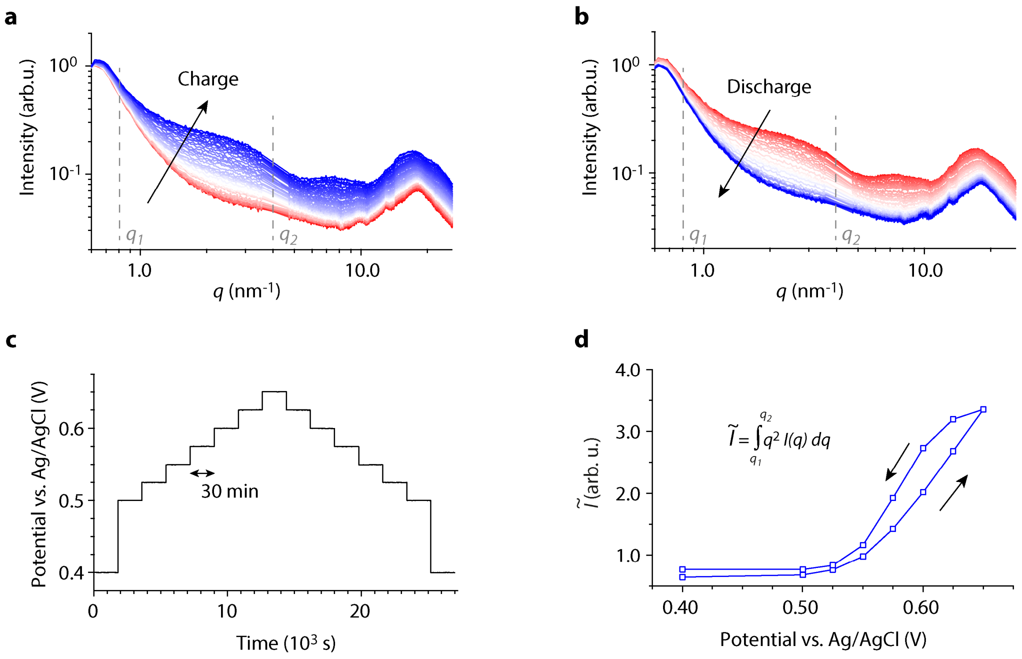

Figure R2.3 (new Supplementary Fig. 16) | SAXS potential dependency during positive and negative cell polarization: (a), scattering intensity versus scattering vector length q during potentiostatic charging in several steps from 0.40 V to 0.65 V vs. Ag/AgCl, as shown in (c). (b), scattering intensity versus scattering vector length q during potentiostatic discharging in several steps from 0.65 V to 0.40 V vs. Ag/AgCl, as shown in (c). (c), WE potential as a function time. (d), Integrated intensity $\tilde{I}$ of reduced experimental in situ SAXS intensities (after subtraction of particle and electrolyte structure factor scattering contributions, equivalent to Fig. 3a and Fig. S11) as a function of the WE potential. The integrated intensity is calculated for the q-regime between q_1 = 0.8 nm^{-1} and q_2 = 4.0 nm^{-1} at the end of each 30 min potential step shown in (c). The integrated intensity shows a clear hysteresis, indicating that I_2 dissolution requires some overpotential during discharge.

As mentioned above, systematic errors (due to the delicate treatment of carbon, iodine/polyiodide, and electrolyte structure factor background) limit the meaningfulness of the in situ SAXS data. Further, the correlation function used for the GRF Z(**x**), i.e. the model as such, might not perfectly fit the real scattering data. Accordingly, differences in contact angles can only be detected with a certain accuracy. Supplementary Figure 11 shows modelled scattering intensities using the three different contact angles δ= 45°, 67.5°, and 90°. Given the severe deviations between model and experiment and the systematic experimental errors smaller δ variations make hardly any sense. Within the (limited) accuracy we did not detect differences in contact angles during charge and discharge. Please note the negligible

differences in the SAXS intensiy shapes during charge and discharge in Fig. R2.2a-b. Alternative correlation functions for the GRF Z(**x**) and the use of other carbons might help to improve the sensitivity of the plurigaussian model fit in future. Limitations and sources of error are now discussed in Supplementary Note 1.

Please find hereunder other relatively minor points.

2.3. How critical are the other parameters of the plurigaussian model for the determination of the iodine volume fraction (Fig. S11)? What does Fig. 3c look like if other values are used for lZ and delta?

The I_2 pore occupancies of different l_z and δ are given in Fig. R2.4. The data corresponds to the model SAXS curves shown in Supplementary Fig. 11. A larger l_z parameter leads to generally lower pore occupancies; a decrease in the contact angle gives tentatively higher pore occupancies.

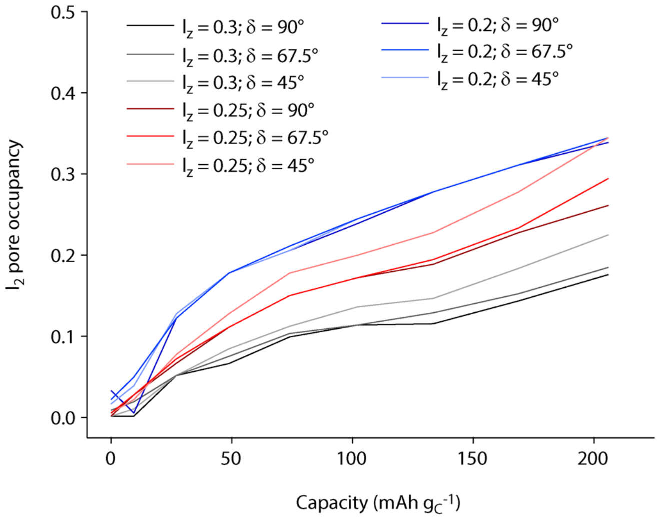

Figure R2.4 : I_2 pore occupancy versus capacity resulting from the SAXS/plurigaussian model fit with different l_z and δ parameters, as shown in Fig. S11.

2.4. How does the surface area determined from nitrogen physisorption compare to the one obtained from SAXS?

The surface area determined by nitrogen adsorption is significantly lower and corresponds to 1763 $m^2\ g^{-1}$ (calculated from the QSDFT method). The SAXS surface area of the GRF two-phase model is 3122 $m^2\ g^{-1}$. It is calculated by[1]

$$SSA_{GRF} = \frac{1}{\varphi_C * \rho_{C.skel}} \frac{2^{3/2}}{\pi} \exp\left(-\frac{\alpha^2}{2}\right) \frac{1}{l_{C,Y}} \text{ , where } \frac{1}{{l_{C,Y}}^2} = \frac{1}{6} \int_0^\infty q^2\, f_Y(q)dq\,.$$

We have added this value to Table S2. The higher surface area determined from SAXS is likely induced by narrow pores, which are not accessible to the nitrogen molecule (<0.5 nm) during adsorption. At the same time, a certain degree of closed porosity is possible. An alternative background subtraction in the Porod regime accounting for an additional q^{-2} term induced by density fluctuations of stacked graphene layers has been suggested by Ruland et al[2]. This would indeed lower the SAXS surface area and be accurate for a certain class of activated carbons. Yet, given the extremely small and disorderly arranged pores of our investigated carbon[3] (known from TEM and a nearly absent 002 XRD peak) an additional q^{-2} term (due to 2D density cavities) at high q seems improper.

---

[1] *C. J. Gommes, Microporous Mesoporous Mater. 257 (2018): 62-78.*
[2] *W. Ruland, J. Appl. Crystallogr. 4, 1 (1971): 70-73.*
[3] *C. Prehal et al., Carbon 152 (2019): 416-423.*

2.5. Insets of Fig. 2e and 2f: Wouldn't one expect a contrast-matching effect, whereby the SAXS signal would initially decrease as solid iodine would bring the electron density of the pore-filling material closer to that of carbon?

We expected this initially. However, given the very high electron density of I_2 with respect to both the carbon and the electrolyte electron density, there is always an increase in the SAXS intensity. This is best seen when considering Eqn. 5 in the methods section and that $\rho_{I2} \gg \rho_C > \rho_{el}$. This qualitatively confirms the formation of solid I_2 (with high density) rather than dissolved I_2 or polyiodides.

2.6. Caption of Fig S10. What is the relevance of parameter alpha for field Z?

This typing error has been fixed.

2.7. Page 5, lines 4-6: Doesn't the attenuation depend on the total amount of iodide, iodine, etc. over the optical path of the x-ray, independently of where these species are positioned?

Yes, this is true. However, since we cut a hole of 2 mm in diameter in the counter electrode (CE) and the separator (Fig. R2.5), the accumulation of iodine and polyiodides in the working electrode (WE) can be detected by the X-ray attenuation in transmission mode.

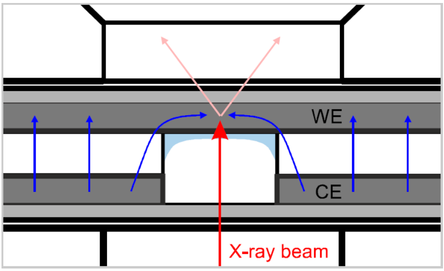

Figure R2.5: Sketch of cell assembly inside the in situ SAXS cell with a 2mm hole cut into CE and separator. Except of the mass ratios, the cell assembly is similar to previous experiments[1].

2.8. In many instances through the paper, the unit of the lengths that enter the plurigaussian model is not specified (e.g. Table S2, Captions of Figs. S10 and S11). Please correct that.

This has been corrected in the revised manuscript.

2.9. Page 15, Eq. 9: I believe alpha and beta have been swapped in the integration limits.

Indeed alpha and beta have been swapped. This has been changed in the revised manuscript.

2.10. Page 15, Eq. 10. Unless I am mistaken, parameter beta is set to infinity for all the models used in the paper. If that is the case, I suggest not mentioning beta at all, and showing only the (simpler) equations in the limit of beta equal infinity.

This has been changed in the revised manuscript.

[1] *C. Prehal et al., Phys. Chem. Chem. Phys. 19 (2017): 15549.*

Reviewer #3:

This work proposes the reaction mechanism during iodine deposition in microporous carbon electrode and suggests that reducing the concentration of I^- in the electrolyte assists the formation of more I_2 and hence mitigates the self-discharge of carbon in the iodide based electrolyte. The mechanism proposed in this study is interesting and the characterization supports well the mechanism proposed.

We thank Reviewer 3 for the constructive comments, which helped raising the quality of the manuscript. We have added new electrochemical data that quantify rate capability, Coulombic efficiency, and cycle life. Further, we discuss the main advantages of aqueous iodine energy storage in more detail.

Iodine energy storage represents a sustainable, cost-effective, and environmentally friendly energy storage principle. The primary goal of this work was to resolve previous stumbling blocks and clearly show the - mechanistically grounded - way forward, rather than benchmarking performance parameters. Our work corrects the previous belief that charging carbon electrodes aqueous iodide solutions would only result in polyiodides (I_3^- and I_5^-). This would equate to relatively poor energies, in the region of hybrid capacitors. Using in situ Raman and SAXS, we show that solid iodine (I_2) forms to fill the pores to a large extent. Confinement allows us to deposit I_2 persistently with minimal self-discharge yet electrochemically reversible at high rates. Our discovery shows how to achieve high packing densities of I_2 enabling capacities that come close to benchmark Li-ion electrodes. And all this at rates and with a cycle life that surpasses what is possible with Li-ion. In fact, rates are those of hybrid capacitors but the exact performance for redox-electrolyte systems depends also on the operating conditions[1]. For example, as seen from Fig. 4a, assuming a capped specific capacity of about 160 $mAh \cdot g_C^{-1}$, this value is accessible even at ultrahigh rates of 10 $A \cdot g_C^{-1}$ (corresponding with a C-rate of 63C). Even higher capacity values can be obtained, as demonstrated by our work in excess of 400 $mAh \cdot g_C^{-1}$.

However, there are several major concerns:

3.1. The paper suggests that the strategy proposed in this study can also be applied to Li-S battery. It is true that the capacity degrades in the Li-S battery because of the loss of polysulfide during the charge/discharge process. But it is difficult to find how the strategy in this study could help the Li-S battery because of the difference between the charging mechanism of Li-S and iodine battery. The iodine formed in the iodine battery comes from the electrolyte, and will turn back to the iodides during discharge. Controlling the concentration of I- in the electrolyte may assist the I_2 deposition. In Li-S battery, the major strategy is to keep the S species within the electrode. The Li^+ intercalation, instead of S species, contributes to the charge storage in the Li-S battery, which is different from I_2 battery,

It is true, we have mentioned the similarity between the found iodine electrodeposition and Li_2S deposition in Li-S batteries or Li_2O_2 deposition in Li-O_2 batteries, as solid iodine electrodeposition in the carbon nanopores was not considered in most previous works. Mentioning the similarities should point out the conversion-type reaction, where the solid active material is electrodeposited in the nanopore network of a carbon cathode during charging and discharging. The pore occupancy of the active material hence determines achievable capacities and reversibility. Avoiding polysulfide shuttling and polyiodide shuttling is beneficial for Li-S batteries and iodide energy storage, respectively.

We did not intend to deny significant differences between iodine and Li-S energy storage. Further, we did not claim that any of the presented mechanistic strategies to improve iodine electrodeposition are of

[1] *J. Lee et al., Prog. Mater. Sci 101 (2019): 46-89.*

direct use for Li-S batteries. The reaction mechanisms are different - and so are the strategies to avoid polyiodide shuttling in the case of our iodide system and polysulfide shuttling the case of Li-S batteries.

We would also like to point at the capability of the in situ SAXS method to follow morphology evolution of deposits in conversion type batteries at sub-nm to sub-micron resolution, a task which appears elusive with other methods. In the Li-S battery, this concerns both Li_2S and S_8. To what extend this may help fighting polysulfide shuttling is a different story of course.

3.2. The iodine battery works in the way that the ion in the liquid phase transforms to solid-phase I2 in the confined space. The improved I2 pore occupancy improves the capacity, but at the same time, it is at the cost of the rate capability. When more ions transform into the solid phase, the pore will be more blocked and slower ion diffusion can be expected.

An increased I_2 pore occupancy will, at some point, lead to pore blocking, which slows done iodide transport. However, since the iodine nanoclusters have a size of less than 1 nm, the reaction takes place essentially at the carbon/iodine - electrolyte interface. Slow bulk diffusion, like in intercalation-type battery materials can be avoided. In Fig. R3.1 and the new Fig. 4a,b we show the excellent rate capability of the system, even though the iodine pore occupancies and capacities are high. Note that the rates exceed those of typical Li-ion batteries about 1 or 2 orders of magnitude and are comparable to rates in (hybrid) supercapacitors.

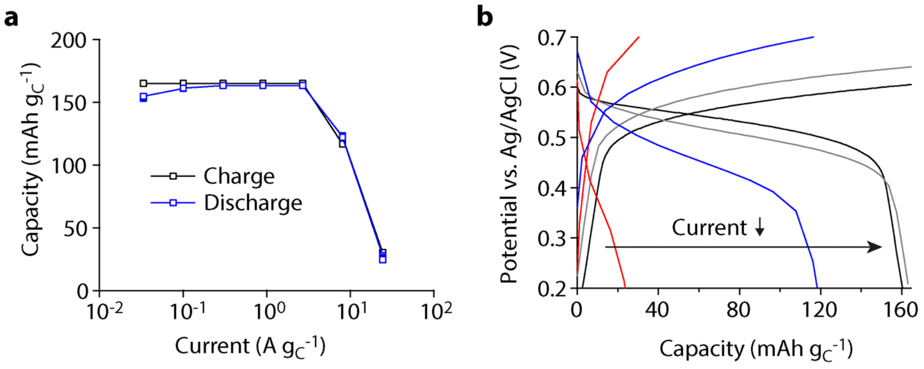

Figure R 3.1 (new Fig. 4a, b) | Rate capability measurements: (a), Specific capacity versus specific current for galvanostatic charge/discharge with capacity limitation of 160 mAh g_C^{-1}. (b), WE potential as a function of capacity for the four highest currents with 24.3 A g_C^{-1} (red), 8.1 A g_C^{-1} (blue), 2.7 A g_C^{-1} (grey), 0.9 A g_C^{-1} (black) of the same galvanostatic charge/discharge measurements.

3.3. It is important to take the amount of electrolyte used in each cell into consideration. As the I-concentration in the electrolyte will decrease during the process, it is not a sustainable process. Or, it is suggested to quantify the ion centration during charging. Also, it is better to show the Coulombic efficiency of the electrode.

Indeed, the total amount of I^- in the system defines the amount of I_2 that can be formed. This is similar to for example Li-S batteries, where the amount of S defines how much polysulfides and Li_2S can be formed. Our work primarily investigates the mechanisms (and their impact on the performance) of the WE. To increase the device performance, a subtle trade-off between electrolyte amount, salt concentration, WE mass, and WE to CE capacity ratio is necessary. This shows in what direction future parameter studies should head to optimize the practical device performance:

- Variation of the electrolyte amount and iodide concentration

- Variation of the fraction of inert supporting electrolyte salt
- Variation of the carbon electrode (pore structure, surface functionalities)
- Variation of the counter-electrode capacity or capacitance

In the discussion section of the revised manuscript, we back-up the performance discussion with capacity values normalized to the mass (or volume) of carbon + electrolyte in the micropores + deposited iodine. This makes the numbers comparable to capacity values of other intercalation-type cathode materials.

We now show the very high Coulombic efficiency over hundreds of cycles in Fig. 4c and Fig. R3.2 below.

3.4. The advantage of the iodine battery is unclear. There is only one electron transfer per I^- if all I^- transfer to I_2, and the capacity of the device depend highly on the pore volume of the AC. The voltage window is very narrow, even narrower than the electrical double layer capacitor, meaning the energy density of the device will be limited. Moreover, the rate capability and cycling stability are not reported here. It is important to claim the advantage of this type of battery.

Besides the mechanistic insights as such, there are several practical aspects that make aqueous iodine energy storage and the current work particularly interesting:

- The very high rate capabilities (power densities) that surpass those of conventional Li-ion battery materials[1] since there are no slow intercalation processes involved. Combined with a high capacity anode (e.g. Zinc metal), this could enable device energy densities in the range of commercial Li-ion batteries and power densities significantly exceeding those of commercial Li-ion batteries.
- All involved materials are abundant, cheap, environmental friendly and non-flammable. This makes iodine energy storage particularly interesting for stationary energy storage - for example in private households, where a somewhat lower energy density compared to Li-ion batteries is acceptable.
- The cycle life is very high and comparable to those of hybrid supercapacitors[2, 3, 4].
- We believe that the mechanistic insights related to iodine electrodeposition also hold for organic electrolytes. Hence, potential windows larger than the water potential window are possible. We have now mentioned the potential use of organic electrolytes in the introduction.

We have added a more detailed discussion to the last part of the manuscript:

"Electrochemical measurements evidence remarkably high capacities. In terms of specific and volumetric capacity solid I_2 (211 mAh g^{-1}, 1040 mAh cm^{-3}) surpasses todays benchmark Li-ion cathode materials such as $LiFePO_4$ (170 mAh g^{-1}, 596 mAh cm^{-3}). To make capacity values comparable to intercalation-type cathode materials the total capacity of the iodine system needs to be normalized by mass/volume of carbon, micropore electrolyte and deposited I_2. An I_2 pore occupancy of 40% in our

---

[1] For comparison let's consider the following work: *An ultrafast battery performing as a supercapacitor: Electrode tuning for high power performance*, Electrochimica Acta 334 (2020) 135587. In this work around 300 mAh/g are reached at C/10, and about 200 mAh/g at 1C. This translates to 200 mA/g, which is significantly lower compared to the 10A/g shown for our system in Fig. R3.1. Our system still reaches 160 mAh/g with a current around 3 A/g. If normalized by mass of carbon + deposited I_2 + remaining electrolyte in the micropores our capacity and current values are lowered by a factor of 0.3, which still results in faster storage than the considered ultrafast battery.

[2] *B. Evanko et al., ACS Energy Letters 2, 11 (2017): 2581-2590.*
[3] *E. Frackowiak et al., ChemSusChem 5, 7 (2012): 1181-1185.*
[4] *J. Lee et al., Prog. Mater. Sci 101 (2019): 46-89.*

carbon with 60% porosity would result in practical capacities of 108.5 mAh g^{-1} and 250 mAh cm^{-3}. We show how to achieve high packing densities at rates significantly exceeding those of Li-ion cathode materials. The absence of slow intercalation processes, enables rates comparable to hybrid supercapacitors."

The new rate capability and cyclability data are shown in Fig. 4a-c. Rate capability and cycle life are very high and comparable to those of hybrid supercapacitors. The rates exceed those achievable with intercalation Li-ion cathode materials.

The Coulombic efficiency is close to 100% for at least several hundred cycles, as shown in Fig. R3.3 (for time reasons, we stopped the cyclability test at 370 cycles, without any sign of degradation).

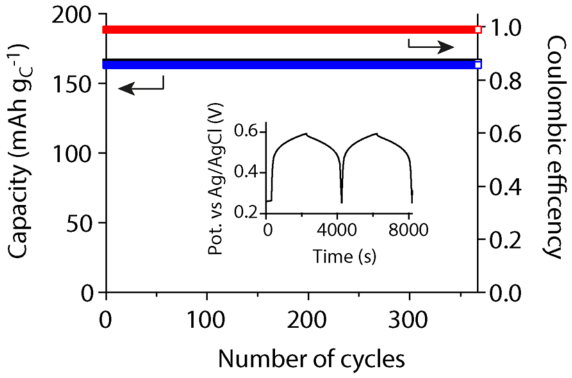

Figure R 3.2 (new Fig. 4c) | Cycle life: Charge/discharge capacity and coulombic efficiency versus number of cycles at a rate of 0.3 A g_C^{-1}. The inset shows the WE potential as a function of time for 2 cycles.

3.5. The battery shows a higher capacity in 0.5 M: 0.5 M NaI: $NaNO_3$ than that in 1M NaI. The improved capacity could be due to complete transformation from I^- to I_2. But it seems that the self-discharge becomes worse as the capacity drops faster in 0.5 M: 0.5 M NaI: $NaNO_3$ electrolyte.

The improved capacity stems from a (more) complete conversion of I^- to I_2. The self-discharge seems to be slightly higher for the 0.5 M NaI / 0.5 M $NaNO_3$ electrolyte. However, given the very flat decay for times beyond 16 h, the 0.5 M NaI / 0.5 M $NaNO_3$ electrolyte enables higher specific capacities for most practical time scales. We plan to carry out a more detailed parameter study related to the device performance in the near future.

REVIEWERS' COMMENTS:

Reviewer #2 (Remarks to the Author):

Dear Editor,
Dear authors,

The authors have thoroughly addressed all the points I raised in my first report. In particular, the procedure by which the volume fraction of the iodine crystals is determined from the SAXS data, is now carefully explained. The authors also use the integrated SAXS intensity to calculate the volume fractions of the crystals in a way that is independent of any model, and the values match (with reasonable uncertainly) the electrochemical values, which I find very convincing.

Moreover, new data was also added to the supporting information, which documents hysteretic effects upon loading/unloading of the cell. This hints at strong interaction between the carbon and the crystals. These interactions are the very reason why iodine crystals are formed at all in this context. I agree with the authors that investigating them in detail is beyond the scope of the present paper. The publication of this work will certainly encourage investigations in that direction.

To sum up, this paper is scientifically very interesting, technically sound, and technologically relevant. The existence of confined crystals in equilibrium with the electrolyte is prone to change the way people think of supercapacitors, and of electrochemical energy storage in general. I therefore look forward to seeing this paper published.

As a side comment, I notice that the authors are willing to make the data available on “reasonable” request. Although editorials in Nature Journals regularly advocate Findable Accessible Interoperable Reusable (FAIR) data, I am not sure what your editorial policy is in that matter. It seems to me that the only unbiased data publication consists in making it either unconditionally available or not at all. In any event, I do not understand what a “reasonable” request is.

With kind regards,

Reviewer #3 (Remarks to the Author):

Most of my concerns have been addressed, and I enjoyed reading this paper. Just one more comment on the new Fig. 4c-Cycle life. It is recommended to report the GCD of the first and the last cycle, instead of two random consecutive cycles.

Response to reviewers on the paper entitled

"Persistent and Reversible Solid Iodine Electrodeposition in Nanoporous Carbons"

Manuscript: NCOMMS-20-13228A

We thank the Reviewers for their positive feedback to which we respond below. The Reviewers' comments are reproduced in italics.

Reviewer #2:

The authors have thoroughly addressed all the points I raised in my first report. In particular, the procedure by which the volume fraction of the iodine crystals is determined from the SAXS data, is now carefully explained. The authors also use the integrated SAXS intensity to calculate the volume fractions of the crystals in a way that is independent of any model, and the values match (with reasonable uncertainly) the electrochemical values, which I find very convincing.

Moreover, new data was also added to the supporting information, which documents hysteretic effects upon loading/unloading of the cell. This hints at strong interaction between the carbon and the crystals. These interactions are the very reason why iodine crystals are formed at all in this context. I agree with the authors that investigating them in detail is beyond the scope of the present paper. The publication of this work will certainly encourage investigations in that direction.

To sum up, this paper is scientifically very interesting, technically sound, and technologically relevant. The existence of confined crystals in equilibrium with the electrolyte is prone to change the way people think of supercapacitors, and of electrochemical energy storage in general. I therefore look forward to seeing this paper published.

As a side comment, I notice that the authors are willing to make the data available on "reasonable request". Although editorials in Nature Journals regularly advocate Findable Accessible Interoperable Reusable (FAIR) data, I am not sure what you policy is in that matter. It seems to me that the only unbiased data oublication conists in making it either unconditionally available or not at all. In any event, I do not understand what a "reasonable" request is.

We thank reviewer 2 for this very positive feedback.

We apologize for the potentially misleading wording; the phrase "reasonable" was used in many Nature articles before, but it was never meant as being conditional. We deleted "reasonable" to state clearly that the data and code will be made unconditionally available upon request.

Reviewer #3:

Most of my concerns have been addressed, and I enjoyed reading this paper. Just one more comment on the new Fig. 4c-Cycle life. It is recommended to report the GCD of the first and the last cycle, instead of two random consecutive cycles.

We thank reviewer 3 for the very positive feedback. We now show the first and last GCD cycle in the inset in Fig. 4c.
